# Analysis of substrate specificity of cytochrome P450 monooxygenases involved in trichothecene toxin biosynthesis

**DOI:** 10.1007/s00253-023-12950-1

**Published:** 2024-01-06

**Authors:** Rosa E. Cardoza, Susan P. McCormick, Natalia Martínez-Reyes, Joaquín Rodríguez-Fernández, Mark Busman, Robert H. Proctor, Santiago Gutiérrez

**Affiliations:** 1 University Group for Research in Engineering and Sustainable Agriculture (GUIIAS), Area of Microbiology, University of León, Ponferrada, 24400 Spain; 2 Agricultural Research Service, Mycotoxin Prevention and Applied Microbiology Research Unit, USDA, National Center for Agricultural Utilization Research, 1815 N University St, Peoria, IL 61604 USA; 3 Area of Biochemistry and Molecular Biology, University of León, Ponferrada, 24400 Spain

**Keywords:** Cytochrome P450 monooxygenases, Trichothecene biosynthesis, Substrate specificity, Gene deletion, Gene expression, Evolutionary relationships

## Abstract

Trichothecenes are a structurally diverse family of toxic secondary metabolites produced by certain species of multiple fungal genera. All trichothecene analogs share a core 12,13-epoxytrichothec-9-ene (EPT) structure but differ in presence, absence and types of substituents attached to various positions of EPT. Formation of some of the structural diversity begins early in the biosynthetic pathway such that some producing species have few trichothecene biosynthetic intermediates in common. Cytochrome P450 monooxygenases (P450s) play critical roles in formation of trichothecene structural diversity. Within some species, relaxed substrate specificities of P450s allow individual orthologs of the enzymes to modify multiple trichothecene biosynthetic intermediates. It is not clear, however, whether the relaxed specificity extends to biosynthetic intermediates that are not produced by the species in which the orthologs originate. To address this knowledge gap, we used a mutant complementation-heterologous expression analysis to assess whether orthologs of three trichothecene biosynthetic P450s (TRI11, TRI13 and TRI22) from *Fusarium sporotrichioides*, *Trichoderma arundinaceum*, and *Paramyrothecium roridum* can modify trichothecene biosynthetic intermediates that they do not encounter in the organism in which they originated. The results indicate that TRI13 and TRI22 could not modify the intermediates that they do not normally encounter, whereas TRI11 could modify an intermediate that it does not normally encounter. These findings indicate that substrate promiscuity varies among trichothecene biosynthetic P450s. One structural feature that likely impacts the ability of the P450s to use biosynthetic intermediates as substrates is the presence and absence of an oxygen atom attached to carbon atom 3 of EPT.

**Supplementary Information:**

The online version contains supplementary material available at 10.1007/s00253-023-12950-1.

## Key points

• *Trichothecene cytochrome P450 monooxygenases differ in substrate specificity.*

• *Trichothecene 15-hydroxylase (TRI11) has promiscuous substrate specificity.*

• *Fusarium TRI11 modifies evolutionarily divergent biosynthetic intermediates.*

**Supplementary Information:**

The online version contains supplementary material available at 10.1007/s00253-023-12950-1.

## Introduction

Trichothecenes are sesquiterpenoid toxins produced by some fungal species in the fungal classes *Dothideomycetes*, *Eurotiomycetes* and *Sordariomycetes* (Proctor et al. 2018, 2020). Some *Fusarium* trichothecene analogs are among the mycotoxins that pose the greatest threat to food and feed safety, and some *Stachybotrys* trichothecene analogs have been linked to human disease symptoms caused by mold growth in damp buildings (Straus 2009). Some *Trichoderma* trichothecenes, such as harzianum A (HA) and 16 hydroxy trichodermin, can contribute to the biological control activity (Malmierca et al. 2012; Ryu et al. 2017). The effects on human activities and/or ecological roles of trichothecene analogs produced by other fungi, including species of *Aspergillus*, *Isaria*, *Microcyclospora*, *Peltaster*, *Trichothecium* and *Paramyrothecium* (formerly *Myrothecium*) remain unclear.

**Fig. 1:**
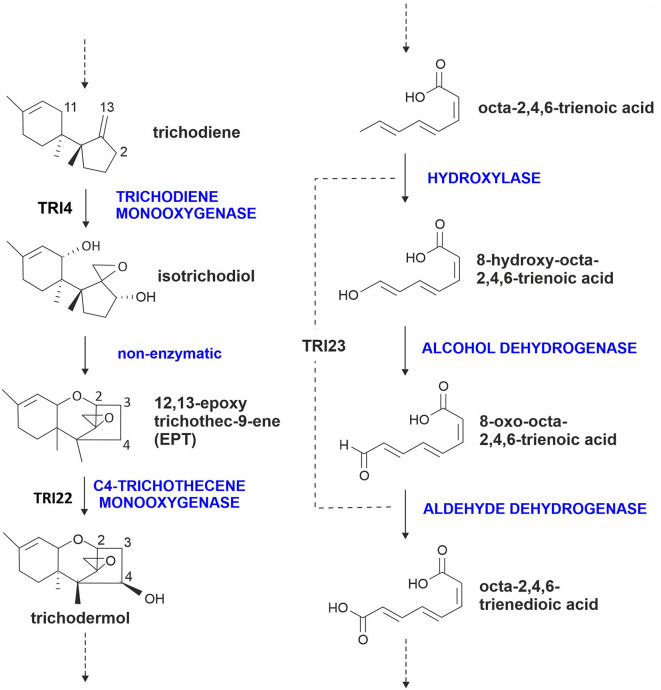
Scheme showing the reactions in harzianum A (HA) biosynthesis catalyzed by cytochrome P450 monooxygenases. Enzyme activities are indicated in black uppercase letters

Over 150 trichothecene analogs have been identified. All of them have the same core structure, 12,13-epoxytrichothec-9-ene (EPT), but vary in the presence, absence, and types of substituents at various positions of the core structure (Fig. S1) (Ueno 1977, 1984; Kimura et al. 1998; McCormick et al. 2011). In *Trichoderma* and *Paramyrothecium*, EPT is formed through the activity of two enzymes: (1) trichodiene synthase (TRI5), a terpene synthase that catalyzes cyclization of the primary metabolite farnesyl diphosphate to form trichodiene; and (2) trichodiene oxygenase (TRI4), a cytochrome P450 monooxygenase (P450) that catalyzes oxygenation of trichodiene at carbon atoms 2, 11 and 13 (C-2, C-11 and C-13) to form isotrichodiol, which can spontaneously cyclize to form EPT (Fig. 1) (McCormick et al. 2011). Much of the known structural variation of trichothecene analogs results from the activities of 12 trichothecene biosynthetic enzymes: the P450s TRI1, TRI11, TRI13, TRI22 and TRI23, which catalyze oxygenation at various positions of EPT (McCormick et al. 2011; Cardoza et al. 2011, 2019); the acyltransferases TRI3, TRI7, TRI16, TRI18 and TRI101, which catalyze esterification of an acetyl or acyl group to some oxygen atoms attached to EPT (Lindo et al. 2019; McCormick et al. 2011; Proctor et al. 2018); the esterase TRI8, which catalyzes deacetylation at C-3 or C-15; and the polyketide synthase TRI17, which catalyzes synthesis of 4–8-carbon acyl moieties that are esterified to the oxygen at C-4 (Proctor et al. 2018, 2020).

Among and within fungal species, structural variation of trichothecene analogs can arise from the presence versus absence of genes encoding TRI enzymes (i.e., *tri* genes). Structural variation can also arise from variability in function of some TRI enzymes. For example, the *Trichoderma* TRI3 catalyzes trichothecene 4-*O*-acetylation (i.e., acetylation of the oxygen atom at C-4) (Lindo et al. 2019; Proctor et al. 2018), whereas *Fusarium* TRI3 catalyzes trichothecene 15-*O*-acetylation (McCormick et al. 1996). Likewise, in most trichothecene-producing fungi, TRI4-catalyzes only the three oxygenation reactions required for formation of isotrichodiol (Cardoza et al. 2011), but the *Fusarium* TRI4 catalyzes a fourth oxygenation as well, resulting in the formation of isotrichotriol (McCormick et al. 2006b), which can spontaneously cyclize to 3-hydroxy EPT (isotrichodermol). Sequence-based phylogenetic analyses of TRI orthologs provide evidence for which trichothecene biosynthetic activities are likely to be ancestral and which are likely to be derived (Proctor et al. 2018). There is also evidence that some of the enzymes have retained low levels of the ancestral activity after their functions have changed. For example, the predominant activity of TRI3 in *Fusarium* species is trichothecene 15-*O*-acetylation, which is considered a derived activity, but the enzyme has retained low levels of the ancestral trichothecene 4-*O*-acetylation activity under some conditions (Tokai et al. 2008). Conversely, the three ancestral activities of TRI4 (i.e., trichodiene 2, 11 and 13-oxygenation) predominate in *Paramyrothecium roridum*, but low levels of the fourth (derived) activity (i.e., trichodiene 3-oxygenation) occur under some conditions (Trapp et al. 1998; Lindo et al. 2019). Functional variation is also evident from the ability of some TRI enzymes to use multiple substrates to catalyze the same trichothecene biosynthetic reaction (Kimura et al. 2007; McCormick and Hohn 1997; McCormick et al. 2011).

In contrast to individual TRI orthologs acquiring new functions, some dissimilar TRI enzymes have convergently evolved to catalyze the same trichothecene biosynthetic reaction. For example, there are two dissimilar P450s that catalyze trichothecene 4-hydroxylation among trichothecene producing fungi: TRI22 in most trichothecene producers but TRI13 in trichothecene-producing *Fusarium* species (Brown et al. 2002; Cardoza et al. 2011; Proctor et al. 2018). However, TRI13 and TRI22 use different substrates and, therefore, synthesize different products. Analysis of *T. arundinaceum* indicate that TRI22 catalyzes 4-hydroxylation of EPT to form 4-hydroxy EPT (trichodermol) (Fig. 1), whereas analysis of *Fusarium sporotrichioides* indicates that TRI13 catalyzes 4-hydroxylation of 3,15-diacetyl EPT (calonectrin) to form 3,15-diacetyl-4-hydroxy EPT (3,15-diacetoxyscirpenol) (Fig. S2) (Brown et al. 2002). Although TRI13 can also use other intermediates that occur later in the trichothecene biosynthetic pathway, it is not known whether TRI13 can use EPT as a substrate or whether TRI22 can use 3,15-diacetyl EPT or other *Fusarium* 3-hydroxy EPT derivatives as a substrate.

Knowledge of functional variability of TRI enzymes has been acquired from characterization of individual enzymes by gene deletion and/or heterologous expression analyses combined with chemical analyses. Within the context of trichothecene biosynthesis, we are not aware of systematic analyses to assess functional variability of enzymes that represent a range in how closely related they are to one another in terms of evolutionary distance and function. Therefore, the objective of the current study was to compare the activities of three trichothecene biosynthetic P450s (TRI11, TRI13 and TRI22) by attempting to genetically complement *tri13* and *tri22* deletion mutants of *F. sporotrichioides* and *T. arundinaceum*, respectively, with *tri11*, *tri13* and/or *tri22* orthologs from various fungal species. We selected P450s for this analysis because of their critical roles in generating structural diversity of trichothecenes and, although they belong to the same enzyme class, they can have markedly different evolutionary histories. The *T. arundinaceum* and *P. roridum* TRI22 orthologs examined in this study were selected to represent distantly related orthologs of the same enzyme; *F. sporotrichioides* TRI13 was selected because TRI13 and TRI22 have different evolutionary histories, belong to different P450 families and share only 23% amino acid sequence identity, but they both catalyze trichothecene 4-hydroxylation; and *F. sporotrichioides* TRI11 was selected because TRI11 and TRI22 also have markedly different evolutionary histories and catalyze different trichothecene hydroxylation reactions.

The results of the current study revealed that only orthologs of *tri22* complement the lack of HA production in a *T. arundinaceum tri22* deletion mutant and, therefore, that other P450s examined (TRI11 and TRI13) cannot compensate for the absence of TRI22 in the mutant. This in turn indicates that the P450s have strict substrate requirements and/or oxygenation activities. The results also revealed that *T. arundinaceum* has another oxygenase enzyme(s) that can modify EPT to form metabolites that have not been detected in this species prior to this study. Finally, examination of the antifungal activity of various transformants of the *tri22* mutant provides additional evidence that HA production contributes to the biological control activity of *T. arundinaceum*.

## Materials and methods

### Strains used and growth conditions

*Trichoderma arundinaceum* IBT 40837 (Ta37) was used as the wild-type strain in the present study. Wild-type field strains of *Fusarium longipes* (strain NRRL 20695) and *F. graminearum* (strain PH-1) were used as sources of *tri11* orthologs, and a wild-type field strain of *F. sporotrichioides* (strain NRRL 3299) was used as a source of a *tri13* ortholog. The *F. sporotrichioides tri13* deletion (Δ*tri13*) mutant, strain ΔFsTRI13.10, which was generated in a previous study (Brown et al. 2002) was used in the complementation experiments. The wild-type *F. sporotrichioides* strain produces T-2 toxin, whereas the Δ*tri13* mutant produces 4-deoxy T-2 toxin (Brown et al. 2002). A wild-type field strain of *Paramyrothecium roridum* (strain NRRL 2183) was used as a source of the *tri22* gene. Finally, *Rhizoctonia solani* (strain R43), a highly virulent phytopathogen, obtained from the “Pathogens and Antagonists culture collection” (University of León, Spain) (PAULE) under the number PAULER006, was used in the antifungal assays.

*Trichoderma* strains were maintained on potato dextrose agar medium (PDA), which was prepared from PDB broth (Becton Dickinson Co.) amended with 2% agar (Oxoid Ltd.). Sporulation of *Trichoderma* strains occurred during growth on PDA at 28 °C in the dark for 7 days. *R. solani* was grown on PDA medium at 28 °C for 5–7 days. *Fusarium* strains were grown and sporulated on CMD medium (0.1% yellow corn meal, 0.5% PDB, 2% Agar) in an incubation chamber at 21 °C, with a photoperiod of 16 h light/ 8 h dark for two weeks. *P. roridum* was grown on CMD_ULE (5% of yellow cornmeal, 0.5% PDB, 2% Agar) at 28 °C in the dark for two weeks.

### Genetic nomenclature

In this study, we used *Trichoderma* genetic nomenclature regardless of gene origins. Thus, wild-type gene names consist of three lowercase and italicized letters (e.g., *tri22*), deleted gene names are the same as wild-type gene names except that they are preceded by an uppercase Greek letter delta (e.g., Δ*tri22*), and protein names consist of three uppercase and unitalicized letters (e.g., TRI22).

### Phylogenetic analysis

The predicted amino acid sequences of TRI11, TRI13 and TRI22 orthologs from trichothecene-producing fungi, and more distantly relatedhomologs from trichothecene-nonproducing fungi were aligned using MUSCLE program as implemented in MEGA version 10.2.6 (Kumar et al. 2018). The alignment was then subjected to a maximum likelihood analysis (ML) by the IQ-Tree v. 1.6.7 software (Nguyen et al. 2014). Branch support was determined by bootstrap analysis based on 1,000 pseudoreplicates. Visualization of the phylogenetic tree was carried out with FigTree v1.4.4 (http://tree.bio.ed.ac.uk/).

### Plasmid construction

#### Construction of *T. arundinaceum tri22* gene deletion plasmid

**Fig. 2:**
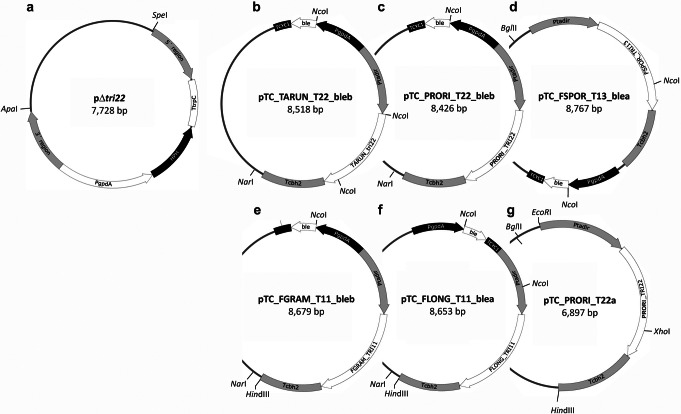
Plasmids designed for the current study: **a** plasmid pΔ*tri22*, **b** pTC_TARUN_T22_bleb, **c** pTC_PRORI_T22_bleb, **d** pTC_FSPOR_T13_blea, **e** pTC_FGRAM_T11_bleb, **f** pTC_FLONG_T11_blea, and **g** pTC_PRORI_T22a. The genetic elements included in these plasmids are TARUN_TRI22, *Trichoderma arundinaceum tri22* exon-intron region; PRORI_*tri22*, *Paramyrothecium roridum tri22* exon-intron region; FSPOR_TRI13, *Fusarium sporotrichioides tri13* exon-intron region; FGRAM_TRI11, *F. graminearum tri11* exon-intron region; and FLONG_TRI11, *F. longipes tri11* exon-intron region; 5´ region and 3´ region indicate the 5′ and 3′ adjacent regions to the *T. arundinaceum tri22* exon-intron region, respectively; PgpdA, promoter region of the glyceraldehyde-3-phosphate dehydrogenase gene from *Aspergillus nidulans*; *HPH*, *Escherichia coli* hygromycin resistance gene coding region; TtrpC corresponds to the *Aspergillus nidulans trpC* terminator region; ble, bleomycin/phleomycin resistance gene coding region from *Streptoalloteichus hindustanus*; TCYC1, terminator region of the *Saccharomyces cerevisiae CYC1* gene; Ptadir, promoter region of the *Trichoderma harzianum tadir* gene; and Tcbh2 indicates, transcriptional terminator of the *Trichoderma reesei* cellobiohydrolase 2 encoding gene

Regions adjacent to *T. arundinaceum tri22* were PCR amplified using 5´-phosphorylated oligonucleotides TRI22_5r_F_BamHI / TRI22_5r_R_SmaI and TRI22_3r_F_SmaI / TRI22_3r_R_SalI (Table S1) to obtain the fragments corresponding to the 5´ (1,013 bp) and 3´  (1,048 bp) regions, respectively. Both fragments were ligated to pBluescript KS + vector (Stratagene) previously digested with *Eco*RV and dephosphorylated with alkaline phosphatase (Thermo Scientific), to get pBT22-5R (3,974 bp) and pBT22-3R (4,009 bp), respectively. pBT22-3R was then digested with *Bam*HI-*Sma*I and ligated with the 1,013 bp fragment isolated from pBT22-5R by digestion with *Bam*HI-*Sma*I, which corresponds to the 5´ region adjacent to *tri22* gene. The resulting plasmid, pBtri22-3r5r (5,013 bp) was linearized with *Sma*I, dephosphorylated with alkaline phosphatase (Thermo Scientific), and ligated to a 2,710 bp fragment containing the hygromycin resistance cassette, which was isolated from pAN7-1 (Punt et al. 1987) by digestion with *Ecl*136II-*Hin*dIII, and treated with Klenow fragment (Thermo Scientific), to finally obtain plasmid pΔ*tri22* (7,728 bp), which includes the hygromycin resistance cassette in the opposite orientation to the sense of *tri22* transcription (Fig. 2a).

#### Construction of *tri* gene overexpression plasmids

To overexpress *tri* selected genes in the *T. arundinaceum tri22* deletion mutant, the coding region of each gene was placed between a 1,129-bp fragment of the 5’-flanking region (promoter) of the *Trichoderma harzianum tadir* gene (formerly *tss1* gene, GenBank accession XM_024914986) and a 1,036-bp fragment of the 3’-flanking region (terminator) of the *Trichoderma reesei cbh2* gene (GenBank accession AF302657). Both the *tadir* promoter and *cbh2* terminator sequences were contained in plasmid pTAchb (Cardoza et al. 2015). In multiple previous studies, constructs consisting of the *tadir* promoter, a *tri* coding region, and the *cbh2* terminator drove high levels of expression of the *tri* genes when the constructs were transformed into *T. arundinaceum* (Cardoza et al. 2015, 2019; Lindo et al. 2019). The *T. arundinaceum tri22* (1,580 bp), *P. roridum tri22* (1,558 bp), *F. sporotrichioides tri13* (1,833), *F. graminearum tri11* (1,741 bp), and *F. longipes tri11* (1,715 bp) were amplified from the genome of the respective fungi using oligonucleotide pairs TARUN_T22_ATG/TARUN_T22_end, PRORI_T22_ATG / PRORI_T22_end, FSPOR_T13_ATG / FSPOR_T13_end, FGRAM_T11_ATG / FGRAM_T11_end, and FLONG_T11_ATG / FLONG_T11_end, respectively (Table S1), and Q5 high-fidelity DNA polymerase (New England Biolabs). The resulting amplified fragments were phosphorylated with polynucleotide kinase (Fermentas), and, after gel purification, the fragments were ligated to the pTAcbh plasmid (Cardoza et al. 2015) linearized with *Nco*I, filled with Klenow fragment, and dephosphorylated. The resulting plasmids, pTC_TARUN_tri22a (6,919 bp), pTC_PRORI_TRI22a (6,897 bp), pTC_FGRAM_TRI11a (7,080 bp), and pTC_FLONG_TRI11a (7,054 bp) were digested with *Eco*RI, filled with Klenow fragment, and dephosphorylated. Plasmid pTC_FSPOR_TRI13a was digested with *Ehe*I and dephosphorylated. The resulting linearized plasmids were finally ligated to the 1,591 bp fragment containing *ble* (bleomycin/phleomycin resistance) cassette, which was isolated from pJL43b1 (Gutiérrez et al. 1997), by digestion with *Hin*dIII, filled with Klenow, and digested with *Ecl*136II. The final plasmids, pTC_TARUN_T22_bleb (8,518 bp), pTC_PRORI_T22_bleb (8,426 bp), pTC_FGRAM_T11_bleb (8,679 bp), pTC_FLONG_T11_blea (8,653 bp), and pTC_FSPOR_T13_blea (8,767 bp), (Fig. 2b-f) were linearized with *Nar*I, *Nar*I,, *Hin*dIII, *Hin*dIII, and *Bgl*II, respectively, prior their transformation into protoplasts of the *T. arundinaceum tri22*-deleted mutant, or *F. sporotrichioides tri13* deleted mutant when indicated.

### Fungal transformation

#### *Trichoderma* transformation

Protoplast transformation procedures used for *T. arundinaceum tri22* gene deletion and overexpression studies were as described previously (Proctor et al. 1999; Cardoza et al. 2006), using hygromycin or phleomycin resistance as the selection markers.

#### *Fusarium sporotrichioides* Δ*tri13* transformation. Growth and protoplasts formation

Thirty mL of CM medium were inoculated with a spore suspension prepared from *F. sporotrichioides* Δ*tri13* (4.26 × 10^5^ spores/mL) and incubated at 28 °C, 250 rpm for 15 h. Mycelium from 20 mL of the resulting culture (loose mycelium without pellets formation), was collected by filtration through Nytal® filters (30 μm pore diameter), washed three times with 0.7 M NaCl, and resuspended in 20 mL NaCl, containing a mixture of lytic enzymes (lysing, 7.5 mg/mL; driselase, 25 mg/mL; chitinase, 0.1 mg/mL). The mixture was incubated at 28 °C, 80 rpm, for 18 h, resulting in a suspension of 5 × 10^7^ protoplasts/mL. Transformation. A co-transformation procedure was carried out using plasmids pGEM-NotI (6,350 bp) (Proctor et al. 2008), conferring geneticin resistance, and pTC_PRORI_T22a (6,897 bp) (Fig. 2g), the latter designed for overexpression of *P. roridum tri22* gene as described above. Plasmids were linearized with *Hin*dIII and *Bgl*II, respectively. Transformation was carried out as described previously (Proctor et al. 1999), using 150 µg/mL, 200 µg/mL, and 300 µg/mL of geneticin (G418, Sigma Aldrich), in the regeneration medium (Base medium: 27.3% sucrose, 0.1% yeast extract, 0.1% NZ amine, and 1.6% Agar; Top medium: 1% Agar in MilliQ water). To confirm the transformation procedure was successful, putative transformants were first grown on PDA without antibiotic and later on PDA amended with 350 µg/mL geneticin.

### Metabolite detection and quantification by high-pressure liquid chromatography (HPLC) and gas chromatography-mass spectrometry (GC-MS)

#### HA quantification by HPLC

HA was detected and quantified from the different strains analyzed in the present work as described previously (Cardoza et al. 2022b). In brief, strains were grown in 250 mL flasks containing 50 mL of complex-malt broth (CM) [0.5% malt extract (Cultimed, Panreac Applichem), 0.5% yeast extract (Difco, Becton Dickinson), 0.5% glucose (Panreac Applichem)], inoculated with 2 × 10^6^ spores/mL and incubated for 24 h at 28 °C and 250 rpm in an orbital shaker. Then, 10 mL of this preinoculum was transferred to a new 250 mL flask containing 50 mL of potato-dextrose broth (PDB) (Difco, Becton Dickinson), and incubated at 28 °C and 250 rpm for 48 h. HA was purified and quantified from the culture supernatants as described by Cardoza et al. (2011). Quantification of HA was carried out by comparison of the 24 min peak area against a calibration curve made with known amounts of purified HA. The 24 min peak is characteristic for this compound in the previously described conditions (Cardoza et al. 2011). Each quantification was performed in two independent biological replicates.

#### Metabolite detection by GC-MS

We used GC-MS to detect trichothecenes (e.g., trichodermol, EPT, isotrichodermin, 15-decalonectrin, calonectrin and 3,15-diacetoxyscirpenol (Cardoza et al. 2011; Proctor et al. 2018), as well as other secondary metabolites (e.g. aspinolides, deoxysambucinol, sambucinol) and fatty acids. Strains were grown in liquid yeast extract-peptone-dextrose (YEPD) cultures [5% glucose, 0.1% yeast extract, 0.1% peptone (Difco, Becton Dickinson)] (20 mL YEPD in 50 mL Erlenmeyer flasks) at 200 rpm and 28 °C. After 7 days, cultures were extracted with 3 mL of ethyl acetate, and concentrated extracts were injected into a Hewlett Packard 6890 gas chromatograph fitted with a HP-5MS column (30 m, 0.25 mm, 0.25 μm) and coupled with a 5973-mass detector as previously described (Cardoza et al. 2022a). Compound identifications were based on comparisons of mass spectral fragmentation with a mass spectral library of purified standards.

#### Feeding experiments

Precursor feeding experiments were done with three trichothecene biosynthetic intermediates: (1) calonectrin, which is produced by *F. sporotrichioides* and is converted to 3,15-diacetoxyscirpenol via TRI13-catalyzed 4-hydroxylation; (2) isotrichodermin, which is also produced by *F. sporotrichioides* and is converted to 15-decalonectrin via TRI11-catalyzed 15-hydroxylation; and (3) EPT, which is produced by *T. arundinaceum* and is converted to trichodermol via TRI22-catalyzed 4-hydroxylation. In feeding experiments with calonectrin and isotrichodermin, transformants of *T. arundinaceum* Δ*tri22* mutant with plasmids expressing a *Fusarium* homolog of *tri13* (ΔT22-Fstri13-5 and ΔT22-Fstri13-7) or *tri11* (ΔT22-Fgtri11-1 and ΔT22-Fgtri11-4) were grown in 8 mL liquid YEPD medium (5% glucose, 0.1% yeast extract, 0.1% peptone/L; in 50 mL Erlenmeyer flask) amended with 100 µL of acetone solutions containing 1.5 mg calonectrin or 1.5 mg isotrichodermin, respectively. These cultures were incubated at 28 °C and 200 rpm for 7 days, and then extracted with 8 mL ethyl acetate. The extracts were analyzed by GC-MS. Similarly, in feeding experiments with EPT, transformants of *F. sporotrichioides* Δ*tri13* mutant expressing *P. roridum tri22* were grown for 7 days in liquid YEPD medium (8 mL) amended with 100 µL of an acetone solution containing 1.5 mg EPT. The resulting cultures were extracted and analyzed by GC-MS as described above.

### Trichothecenes and other compounds used in feeding experiments, antifungal assays, and as standards

Sambucinol and deoxysambucinol were isolated from a transformant of the *Fusarium sporotrichioides tri4* UV mutant strain MB5493 (McCormick et al. 1989) expressing *Paramyrothecium roridum tri4* (Trapp et al. 1998). Isotrichodermin was isolated from YEPD cultures of a *F. sporotrichioides* C-15 oxygenase *tri11*-mutant (McCormick and Hohn 1997). 15-decalonectrin was prepared from a*F. sporotrichioides tri3* mutant strain (McCormick et al. 1996). Isotrichodermol was isolated from a *F. sporotrichioides tri101* mutant (McCormick et al. 1999). EPT was prepared by deoxygenation of isotrichodermol (McCormick et al. 1990). 15-hydroxy EPT was prepared by deoxygenation and hydrolysis of 4,15-diacetoxyscirpenol (McCormick et al. 1990). Calonectrin was prepared by treating 15-decalonectrin (isolated from a *Fusarium sporotrichioides tri3* mutant strain (McCormick et al. 1996) with acetic anhydride in pyridine. 3,15-diacetoxyscirpenol was prepared by feeding calonectrin to *Fusarium verticillioides* strain M-3125 (McCormick et al. 2006a). Aspinolides were isolated from a *tri5* mutant of *T. arundinaceum* (Malmierca et al. 2015).

### Antifungal assays

Antifungal assays were performed using cellophane membranes (Shenzhou Pengyu Trading Co., Ltd) as described previously (Malmierca et al. 2013). *R. solani* was grown, after the membrane removal, for 7 days at 28 °C. Diameter of colonies of each plate were measured and photographed after that time. Percentages of radial growth inhibition were calculated as previously described (Cardoza et al. 2007; Royse and Ries 1978).

To analyze the effect of deoxysambucinol and sambucinol on *R. solani* growth, 60 µL of each of these purified compounds were placed, at concentrations from 10 mg/mL to 0.0025 mg/mL, into a 7 mm hole performed in the center of modified-PDA plates (1% agar). These plates were maintained at 4 °C for 24 h to allow the diffusion of the added compounds. Then, a plug from a 7-days PDA grown *R. solani* culture was placed inside the hole of each plate, and cultures were incubated for five days at 28 °C. Finally, radial growth inhibition values were calculated as described above.

### Real time qPCR analysis

For qPCR experiments, strains were grown for 48 h. RNAs were purified, and cDNAs synthesized as described previously (Lindo et al. 2018). Note that 48 h is the time point at which previous studies indicate *tri* genes in *T. arundinaceum* exhibit maximum levels of expression under trichothecene production conditions that were also used in the current study (Malmierca et al. 2012). cDNAs were quantified using a Nanodrop ND-1000 (ThermoFisher). qPCR reactions were carried out on a Step One system (Applied biosystems) using the express SYBR green qPCR super-Mix Universal (Invitrogen) as described by the manufacturer, and oligonucleotides designed for analysis of *T. arundinaceum tri22* and α-actin-encoding genes; *F. longipes* and *F. graminearum tri11*; *F. sporotrichioides tri13*, *tri5*, and *tri4*; and *P. roridum tri22* (Table S1). The amplification efficiencies of primer pairs ranged between 92.1% and 109% (Table S1). Finally, the qPCR Ct values were analyzed using the REST©2009 software (Pfaffl et al. 2002) to determine the expression ratio levels as well as the oligo pairs amplification efficiencies. Each measurement was done in triplicate.

### Genome sequencing

The genome sequence from the *T. arundinaceum tri22*-deleted mutant (strain Δtri22.10) was generated using a MiSeq Illumina platform (Illumina, Inc.) and raw sequences were processed with CLC Genomics Work Bench v.22 (Qiagen, Redwood City) as previously described (Proctor et al. 2018). The processed sequence reads of Δtri22.10 mutant strain were submitted to the Sequence Read Archive (SRA) database at the National Center for Biotechnology Information (NCBI) as accession SRR25782766. In addition, sequence reads from the Ta37 wild-type genome were also deposited with the accession SRR25798607.

## RESULTS

### Phylogenetic analysis

**Fig. 3:**
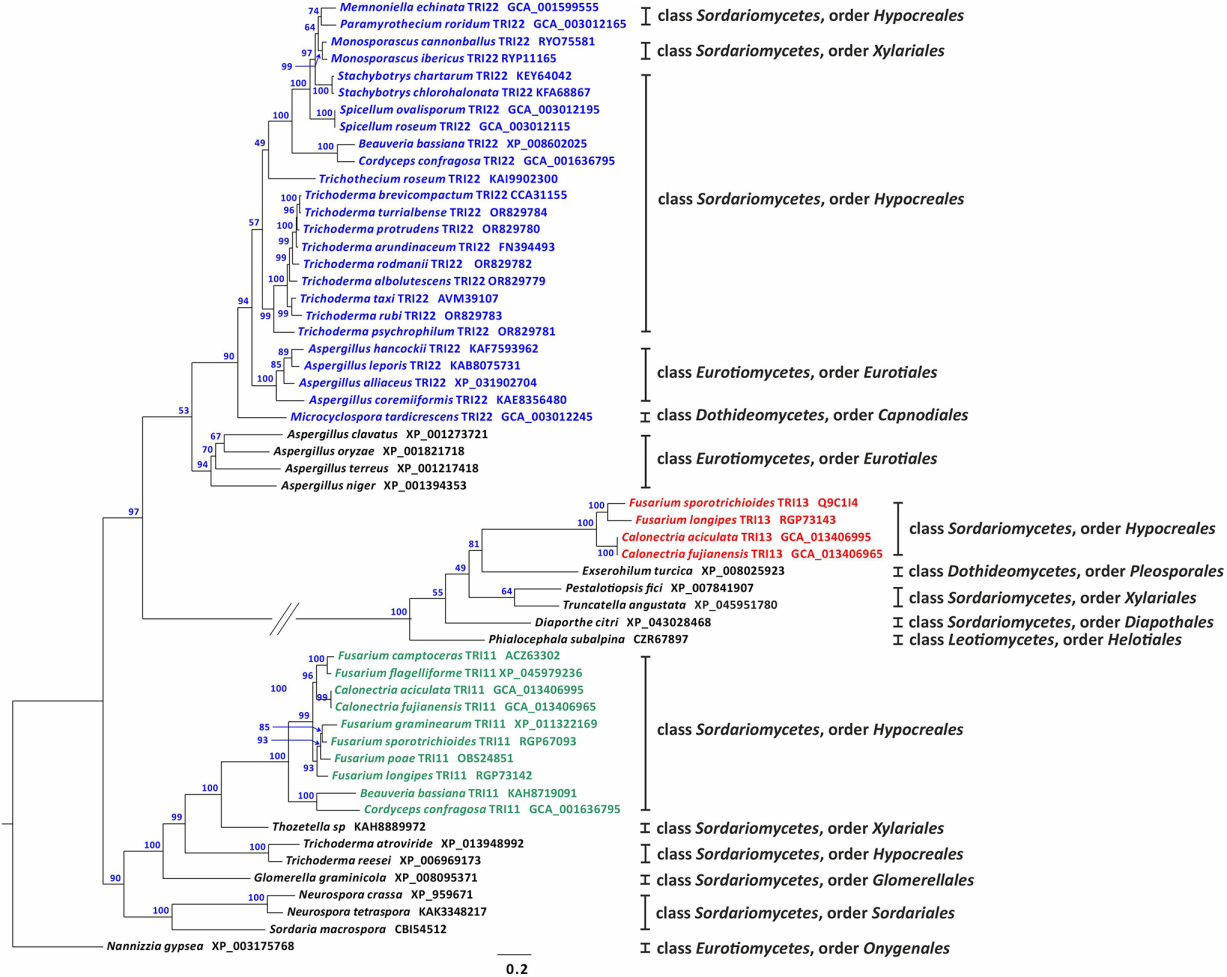
Phylogenetic tree inferred from full-length predicted amino acid sequences of TRI22, TRI11, TRI13 homologs, and *Trichoderma* TRI22-like, *Fusarium* TRI11-like and *Fusarium* TRI13-like encoded proteins. Alignment was subjected to a maximum likelihood analysis, and branch support (values included on each branch) was determined by a bootstrap analysis with 1,000 pseudoreplicates. Blue, red, and green type were used to distinguish TRI22, TRI13 and TRI11 homologs, respectively. Species names written in black type correspond to TRI22-like, TRI11-like and TRI13-like homologs included in this study. The taxonomic class and order for the species included in this study are indicated at the right of the phylogenetic tree. Letter/number designations after taxon names indicate NCBI/GenBank accessions numbers. For a protein sequence obtained from an annotated genome sequences in GenBank, the accession number is for a protein/gene. For protein sequences derived from unannotated genome sequence, the accession is for the genome sequence (= accession numbers with the prefix GCA_) (Sequences used in this analysis were included in the (Supplementary File 1)

The role of the *tri11, tri22, and tri13* genes in trichothecene biosynthesis has been already determined in *Fusarium sporotrichioides, Trichoderma arundinaceum* and *F. sporotrichioides*, respectively. Thus, *Fusarium tri11*-encoded enzyme (TRI11) is responsible for C-15-hydroxylation of isotrichodermin to produce 15-decalonectrin (Alexander et al. 1998), *Fusarium* TRI13 synthesizes 3,15-diacetoxyscirpenol by C-4-hydroxylation of calonectrin (Brown et al. 2002), and *Trichoderma* TRI22 catalyzes C-4 hydroxylation of EPT to produce trichodermol, as deduced from the present work as well as from previous reports (Cardoza et al. 2011). In the current study, to assess the evolutionary relationship between *tri22*/*tri11*/*tri13* homologs we conducted a phylogenetic analysis with TRI11, TRI22, and TRI13 homolog proteins identified from trichothecene-producing fungi and TRI11/TRI22/TRI13-like homologs from trichothecene-nonproducing fungal species, which were retrieved from the GenBank database among those showing the highest scores when using TRI11, TRI22, and TRI13 proteins as a query. In the resulting phylogenetic tree, TRI11, TRI22 and TRI13 homologs grouped into three well-supported clades. One clade included the C-15 hydroxylases TRI11 from *Fusarium, Calonectria, Beauveria and Cordyceps* species, the second clade included the C-4 hydroxylase TRI22 from *Trichoderma, Trichothecium, Spicellum, Aspergillus, Beauveria, Cordyceps, Paramyrothecium, Memnoniella, Monosporascus, Stachybotrys*, and *Microcyclospora*, and the third clade included C-4 hydroxylases TRI13 from *Fusarium* and *Calonectria* species. These clades were more distantly related to one another than they were to TRI11/TRI22/TRI13-like homologs from trichothecene-nonproducing fungi (Fig. 3).

#### Deletion of *T. arundinaceum tri22*

**Fig. 4:**
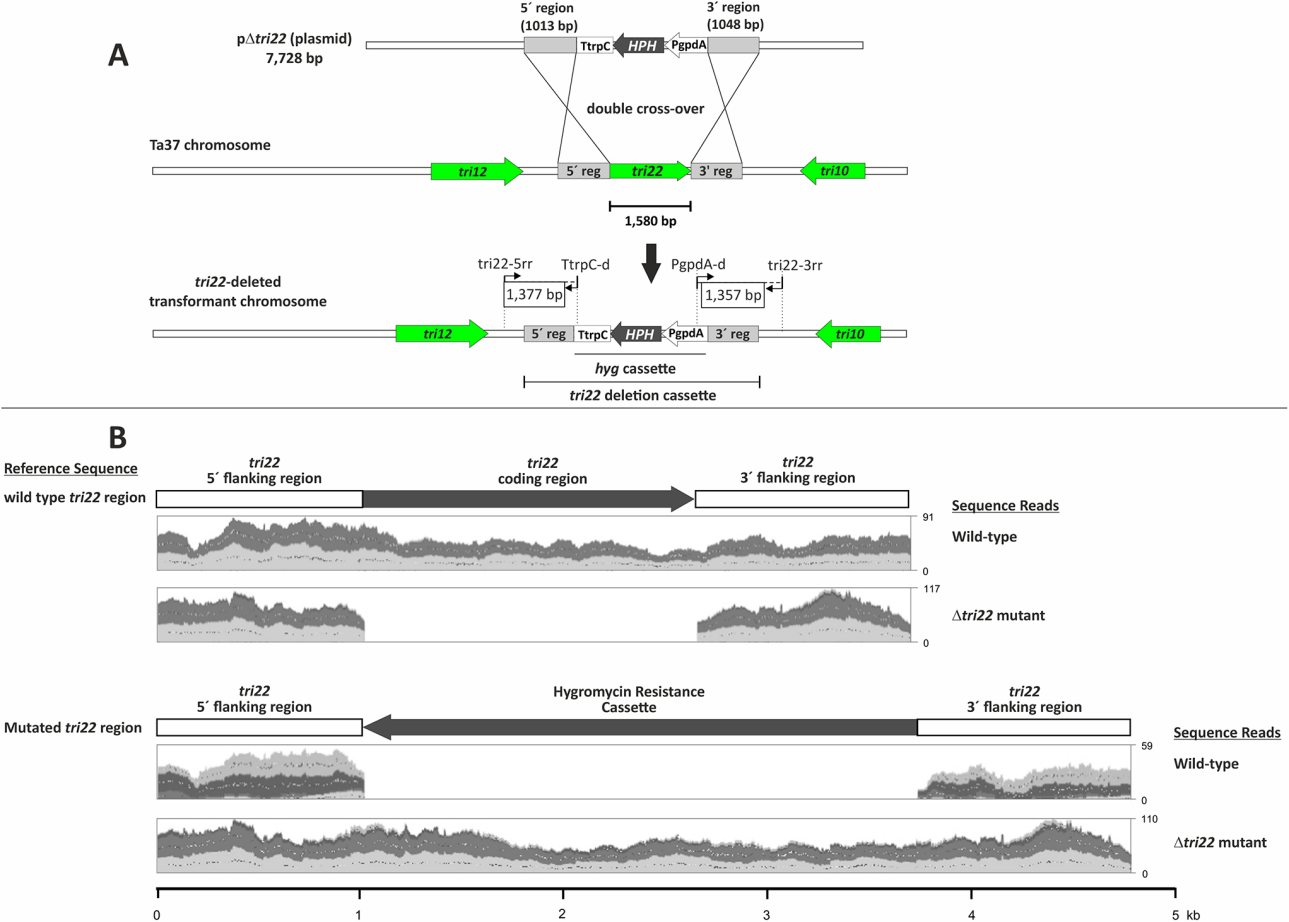
A Strategy used for *tri22* deletion in *Trichoderma arundinaceum* using plasmid pΔ*tri22*. The regions labeled as 1,377 bp and 1,357 bp shown within boxes in the lower panel of (**A**) were amplified by PCR and sequenced to confirm *tri22* deletion. (**B**) Confirmation of deletion of the *tri22* coding region in *Trichoderma arundinaceum* by *in silico* mapping analysis of whole genome sequence reads to reference sequences corresponding to the wild-type *tri22* locus (upper panel) and the predicted *tri22* locus in which the *tri22* exon-intron region was replaced by the hygromycin B resistance cassette (lower panel). For the analysis, the Read Mapping function in CLC Genomics Workbench was used to map MiSeq-generated sequence reads from the wild-type progenitor strain IBT 40837 and the putative *tri22* deletion mutant, strain Δtri22.10. In the upper panel, dark gray arrow indicates the position of the *tri22* exon-intron region, while in the lower panel the dark gray arrow indicates the position hygromycin B resistance cassette. Read mapping results are depicted as uneven, light and dark gray shading within the two rectangles located below each reference sequence. Read coverage is indicated by the numbers to the right of each rectangle. For example, the value 91 to the right the rectangle for wild-type reads mapped to the wild-type reference sequence indicates a maximum coverage of 91 reads

Transformation of the wild-type strain Ta37 with plasmid pΔ*tri22* (Fig. 2a), resulted in 20 transformants that retained hygromycin B resistance after multiple rounds of selection. PCR analyses of these transformants with oligonucleotide pairs corresponding to the 5´- and 3´-regions of the construct expected as result of a double recombination event in the *tri22* genomic region, renders the two bands of 1,377 bp and 1,357 bp expected for the 5´ and 3´extremes of the construct, respectively, in transformants #3, #10 and #17 (Fig. S3). Sequence analysis of these PCR fragments confirmed that *tri22*-deletion occurred as designed (Fig. 4A, Fig. S3) in the three selected transformants. Δtri22.10 mutant was selected for further studies.

To confirm deletion of the *tri22* coding region, we conducted *in silico* mapping of genome sequence reads from the wild-type progenitor strain Ta37 and the putative *tri22* deletion mutant strain Δtri22.10. The reads were mapped to reference sequences consisting of (a) the wild-type *tri22* locus and (b) the predicted sequence of the *tri22* locus in which the *tri22* coding region was replaced by the hygromycin B resistance cassette. Results of the mapping analysis were consistent with replacement of the *tri22* coding region with the hygromycin cassette in the genome of strain Δtri22.10. That is, strain Δtri22.10 (= Δ*tri22*) lacked sequence reads corresponding to the *tri22* coding region, but it had reads corresponding to hygromycin cassette. Further, the read coverage for the cassette was similar to the coverage for the *tri22* flanking regions, a result that is consistent with the presence of a single copy of the cassette. In contrast, the wild-type strain had sequence reads corresponding to the *tri22* coding region but lacked reads corresponding to the hygromycin cassette (Fig. 4B).

**Fig. 5:**
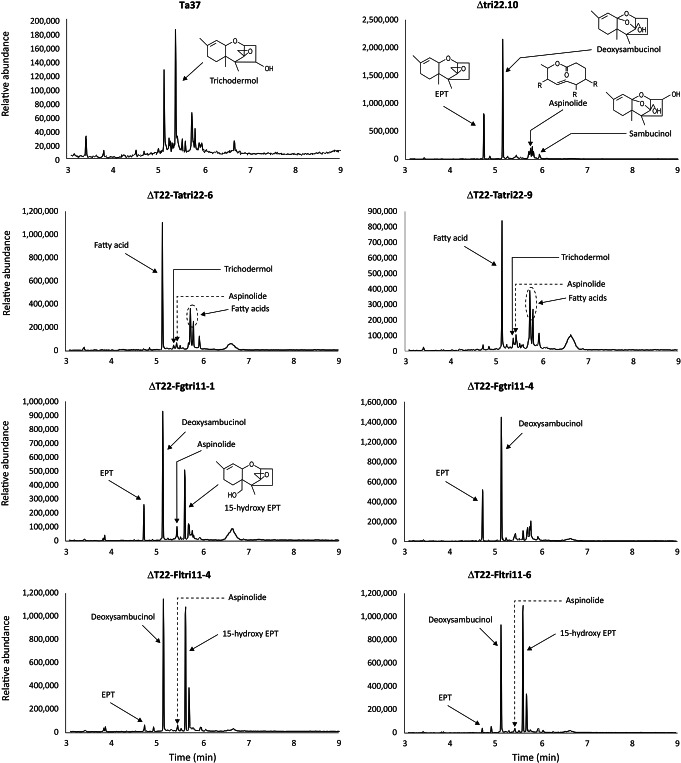
Gas chromatography-mass spectrometry (GC-MS) analysis of extracts of cultures of *Trichoderma arundinaceum*. Ta37, wild-type *T. arundinaceum* strain IBT 40837; *T. arundinaceum tri22* deletion mutant strain Δtri22.10 (= ΔT22); ΔT22-Tatri22-#, Δ*tri22* mutant carrying *T. arundinaceum tri22* expression construct; ΔT22-Fgtri11-#, Δ*tri22* mutant carrying *F. graminearum tri11* expression construct; ΔT22-Fltri11-#, Δ*tri22* mutant carrying *F. longipes tri11* expression construct. Note the scale at the Y-axis differs is some chromatograms

HPLC analysis of culture filtrates indicated that the wild-type progenitor strain produced an average of 246 µg/mL HA in 48-hour PDB culture. Under the same culture conditions, the Δ*tri22* mutant produced an average of 1.5 µg/mL, which represents only 0.6% of wild-type levels (Table 1). In addition, GC-MS analysis revealed that the mutant produced trichothecene (= 12,13-epoxytrichothec-9-ene or EPT), which is the substrate for TRI22 enzyme, as well as aspinolides, deoxysambucinol, and sambucinol (Fig. 5). Interestingly, while aspinolides production has been described in previous studies of *Trichoderma tri*-gene deletion mutants (Izquierdo-Bueno et al. 2018; Lindo et al. 2018; Cardoza et al. 2022b), production of sambucinol and deoxysambucinol by such mutants has not been previously reported.

**Table 1:** Assessment of ability of trichothecene biosynthetic genes encoding cytochrome P450 monooxygenases to complement the *Trichoderma arundinaceum* Δ*tri22* mutant (strain Δtri22.10) using harzianum A (HA) production as an indicator of complementation

Strain	Progenitor strain	Complementing gene	HA Production^b^ µg/mL	Percent
IBT 40837 (wild type)	NA^a^	NA^a^	246.55 ± 8.39	100%
Δtri22.10	IBT 40837	NA^a^	1.52 ± 0.88	0.6%
ΔT22-Tatri22-6	Δtri22.10	*T. arundinaceum tri22*	134.51 ± 2.85	54.5%
ΔT22-Tatri22-9	Δtri22.10	*T. arundinaceum tri22*	124.92 ± 27.3	50.7%
ΔT22-Prtri22-5	Δtri22.10	*P. roridum tri22*	100.19 ± 18.57	40.6%
ΔT22-Prtri22-13	Δtri22.10	*P. roridum tri22*	110.80 ± 9.79	44.9%
ΔT22-Fstri13-5	Δtri22.10	*F. sporotrichioides tri13*	1.44 ± 0.00	0.6%
ΔT22-Fstri13-7	Δtri22.10	*F. sporotrichioides tri13*	1.61 ± 0.25	0.6%
ΔT22-Fgtri11-1	Δtri22.10	*F. graminearum tri11*	2.63 ± 1.34	1.1%
ΔT22-Fgtri11-4	Δtri22.10	*F. graminearum tri11*	1.44 ± 0.08	0.6%
ΔT22-Fltri11-4	Δtri22.10	*F. longipes tri11*	1.39 ± 0.78	0.5%
ΔT22-Fltri11-6	Δtri22.10	*F. longipes tri11*	3.37 ± 1.99	1.4%

Ta- *Trichoderma arundinaceum*, Pr- *Paramyrothecium roridum*; Fs- *Fusarium sporotrichioides*; Fg- *F. graminearum*; Fl- *Fusarium longipes*^**a**^NA indicates not applicable.

^b^ HA production values were derived from analysis of two replicate cultures of each strain. Percent values were calculated based on production in wild-type *T. arundinaceum* strain IBT 40837. HA levels were determined by HPLC analysis of filtrates from 48-hour PDB cultures

### Complementation of *T. arundinaceum* Δ*tri22* mutant

**Fig. 6:**
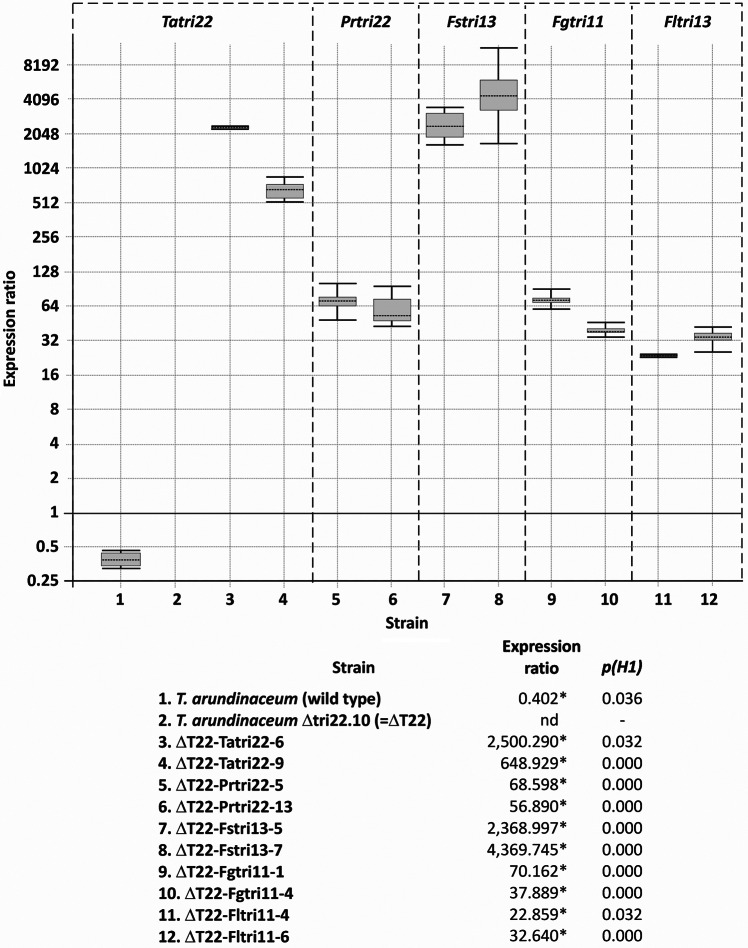
Heterologous expression of cytochrome P450 monooxygenase-encoding genes from *Paramyrothecium roridum* and *Fusarium* species transformed into the *T. arundinaceum tri22* deletion mutant, strain Δtri22.10 (ΔT22). Strains labeled as 1–12 in the upper panel are the same as those listed as 1–12 in the lower panel. Cytochrome P450 monooxygenase genes: *T. arundinaceum tri22* (*Tatri22*) in wild-type *T. arundinaceum* strain (sample 1); ii) in the ΔT22 mutant (sample 2); and iii) in transformants of the latter with plasmid overexpressing *T. arundinaceum tri22* (samples 3–4); *tri22* gene in transformants of ΔT22 expressing *Paramyrothecium roridum tri22* (Prtri22) (samples 5, 6); *tri13* gene in ΔT22 transformants expressing *F. sporotrichioides tri13* (Fstri13) (samples 7, 8); and *tri11* gene in ΔT22 transformants expressing *F. graminearum tri11* (Fgtri11) (samples 9, 10) and *F. longipes tri11* (Fltri11) (samples 11, 12). Values correspond to ratios relative to the expression level of actin gene, which was used as a reference in the current study. Data are from quantitative PCR analysis, and values represent the ratio of expression of each gene relative to the level of expression of *T. arundinaceum* α-actin encoding gene. Calculations and statistical analysis were performed by REST©2009 software (Pfaffl et al. 2002). Statistically significantly different values (*p(H1)* < 0.05) are indicated with an asterisk in the lower part of the figure. nd = not detected

To complement the *tri22* deletion mutant, we transformed the mutant with a plasmid (pTC_TARUN_T22_bleb; Fig. 2b) designed to drive constitutive expression *T. arundinaceum tri22* in transformants. The transformation protocol yielded 22 transformants that retained phleomycin resistance after multiple rounds of selection. In PCR analysis of the transformants with the oligonucleotide primers TARUN_T22_ATG and TARUN_T22_end (Table S1), three transformants (T1, T6, T9) yielded the 1,580-bp amplicon indicative of the presence of the full-length exon-intron region of *T. arundinaceum tri22* (Fig. S4A). HPLC analysis of 48 h PDB cultures of transformants T6 and T9 revealed that they produced markedly higher levels of HA than the Δ*tri22* mutant (Table 1). Production of HA by transformants T6 and T9 was expected since expression of *tri22* was restored in them. Furthermore, although the wild-type progenitor strain (Ta37) produced higher levels of HA than transformants T6 and T9, *tri22* expression in the wild type was much lower compared to the transformants (Fig. 6).

### Complementation with *P. roridum tri22*

*T. arundinaceum* and *P. roridum* TRI22 orthologs, despite their evolutionary distance (Fig. 3) (Proctor et al. 2018) would catalyze the same type of reaction in trichothecene biosynthesis (i.e., trichothecene 4-hydroxylation) and potentially use the same substrate (i.e., EPT) (Cardoza et al. 2011; Proctor et al. 2020). Thus, it is likely that the *P. roridum tri22* ortholog can complement the *T. arundinaceum tri22* mutant. To test this, we transformed the mutant with a plasmid (pTC_PRORI_T22_bleb; Fig. 2c) designed to drive constitutive expression of *P. roridum tri22*. The transformation protocol yielded 28 transformants that retained phleomycin resistance after multiple rounds of selection. In PCR analysis of these transformants with oligonucleotides PRORI_T22_ATG and PRORI_T22_end (Table S1), 11 transformants yielded the 1,558-bp amplicon that was indicative of the presence of the full-length exon-intron region of *P. roridum tri22* (Fig. S4B).

HPLC analysis of filtrates from PDB cultures of two of the transformants (T5 and T13) revealed that they produced markedly higher levels of HA than the *tri22* mutant (Table 1). The increased production of HA in transformants T5 and T13 was consistent with the relatively high levels of expression of the *P. roridum tri22* in the two transformants as determined by qPCR (Fig. 6). These results indicate that *P. roridum tri22* can complement the *T. arundinaceum* Δ*tri22*, indicating that *P. roridum* TRI22 uses the same substrate as *T. arundinaceum* TRI22. Complementation of the Δtri22.10 mutant with the *P. roridum tri22* ortholog resulted in production of lower levels of HA than complementation with the *T. arundinaceum tri22* ortholog. However, the differences were not statistically different.

### Attempted complementation with *F. sporotrichioides tri13*

In order to test whether *Fusarium* TRI13 can catalyze 4-hydroxylation of EPT, we transformed the Δ*tri22* mutant with a plasmid (pTC_FSPOR_T13_ble_a) that was designed to drive constitutive expression of the *F. sporotrichioides tri13* ortholog in transformants (Fig. 2d). The transformation protocol yielded 18 transformants that retained phleomycin resistance after multiple rounds of selection. PCR analysis of the transformants with oligonucleotides FSPOR_TRI13_R1 and Pta (Table S1) yielded the 753-bp amplicon indicative of the presence of the *F. sporotrichioides tri13* in all the transformants (Fig. S4C). Five of these transformants were analyzed for HA production as described above. All five produced levels of HA that were essentially the same as those produced by the Δ*tri22* mutant. HA production results for two of the transformants (T5 and T7) are shown in Table 1.

qPCR analysis of transformants T5 and T7 confirmed that they expressed the *F. sporotrichioides tri13* at relatively high levels (Fig. 6). To confirm that *tri13* expressed in the transformants resulted in a functional TRI13 enzyme, we did a precursor feeding analysis in which calonectrin, the TRI13 substrate in *Fusarium*, was added to YEPD cultures of transformants T5 and T7. Both transformants converted the added calonectrin to 3,15-diacetoxyscirpenol (Fig. S5), at a ratio approaching 100% in transformant T7 and at a lower but significant percentage of conversion in transformant T5. These results indicate that the heterologously expressed *tri13* resulted in a functional protein in both transformants but did not complement the *T. arundinaceum* Δ*tri22* mutant. This in turn indicates that *F. sporotrichioides* TRI13 lacks EPT 4-hydroxylation activity.

### Attempted complementation with *Fusarium tri11* orthologs

To test whether *Fusarium* TRI11 has EPT 4-hydoxylation activity we transformed the *T. arundinaceum* Δ*tri22* mutant with a plasmid designed to drive constitutive expression of *F. graminearum tri11* (pTC_FGRAM_T11_bleb; Fig. 2e) or *F. longipes tri11* (pTC_FLONG_T11_blea; Fig. 2f) in transformants. Each plasmid was transformed separately into the Δ*tri22* mutant, after which we did multiple rounds of selection to obtained 10 transformants for each plasmid that retained phleomycin resistance. We then did PCR analysis using appropriate oligonucleotide combinations to determine whether the phleomycin transformants had a full-length exon-intron region of *tri11*. Five transformants (T1, T2, T4, T6, T7) yielded the 1,905-bp amplicon indicative of the presence of *F. graminearum tri11* (Fig. S4D), and six transformants (T3, T4, T5, T6, T8 and T9) yielded the 1,864-bp amplicon indicative of *F. longipes tri11* (Fig. S4E). HPLC analysis of the 11 transformants indicated that they all produced HA at levels that were essentially the same as those produced by the Δ*tri22* mutant. The HA production for two transformants obtained with each plasmid are presented in Table 1.

We used qPCR to determine whether *tri11* was expressed in selected transformants. This analysis confirmed relatively high levels of *tri11* expression in transformants T1 and T4 carrying the *F. graminearum tri11* and transformants T4 and T6 carrying the *F. longipes tri11* (Fig. 6). We also performed a precursor feeding experiment to confirm the functionality of TRI11 in the selected transformants. In *Fusarium* trichothecene biosynthesis, TRI11 catalyzes 15-hydroxylation of isotrichodermin (3-acetyl EPT) to form 15-decalonectrin (3-acetyl 15-hydroxy EPT) (Fig. S2). Therefore, in the feeding experiment, we added isotrichodermin to YEPD cultures of transformants T1 and T4 carrying the *F. graminearum tri11*. Both transformants converted the added isotrichodermin to 15-decalonectrin (Fig. S6), at a ratio approaching 100%. Together, these results indicate that the *F. graminearum tri11* in transformants T1 and T4 was expressed (Fig. 6) and resulted in a functional enzyme (Fig. S6) but that *tri11* did not complement the *T. arundinaceum* Δ*tri22* mutant (Table 1). Therefore, TRI11 lacks EPT 4-hydroxylation activity.

### Expression of *P. roridum tri22* gene in a *F. sporotrichioides* Δtri13 mutant

In a reciprocal approach to that described above, we tried to determine whether *P. roridum* TRI22 can compensate for the absence of TRI13 in the *F. sporotrichioides* Δ*tri13* mutant. In the current study, we employed a co-transformation approach in which the Δ*tri13* mutant was transformed with two plasmids: one plasmid (pTC_PRORI_T22a; Fig. 2g) carried the *P. roridum tri22* fused to the Ptadir promoter, and the other plasmid (pGen-NotI) carried the geneticin resistance gene as a selectable marker. In PCR analysis of transformants with the primer pair PRORI_T22_ATG/Tcbh2 (Table S1), three transformants yielded the 1,722 bp fragment corresponding to the full-length exon-intron region of *P. roridum tri22* (Fig. S4F).

**Fig. 7:**
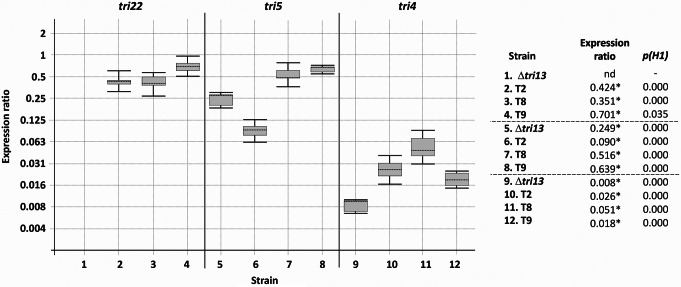
Expression of *tri4, tri5* and *tri22* in three strains T2, T8 and T9 generated by transformation of the *Fusarium sporotrichioides tri13* deletion mutant (Δ*tri13*) with the *Paramyrothecium roridum tri22* ortholog. Values are from quantitative PCR analysis and correspond to ratios of expression of the target gene (*tri4, tri5* or *tri22*) to expression of the *F. sporotrichioides* actin gene. Data were analyzed and statistical significance were calculated as indicated in the legend to Fig. 6. Statistically significant values (*p(H1)* < 0.05) are indicated with an asterisk in the right panel. nd = not detected

In this case, analysis by qPCR of the three selected transformants was carried out from mycelia grown for 7 days on YEPD medium, and results indicated significantly detectable levels of *P. roridum tri22* expression in the analyzed transformants, when compared to the levels reached by α-actin gene (Fig. 7). However, the levels of expression were much lower that those observed when *P. roridum tri22 *was used to complement *T. arundinaceum* Δ*tri22* mutant. These differences in expression can be explained based in the following: (i) the *P. roridum tri22* gene is expressed under the control of a *Trichoderma* promoter region, which might confer lower levels of expression in *F. sporotrichioides* than in *Trichoderma* species, thus conferring a lower level of transcription, and (ii) the media and time point used to grow the *F. sporotrichioides* Δ*tri13*-mutant and the transformants of this mutant expressing *P. roridum tri22* were markedly different to those used for the *Trichoderma* transformants used in this study. *F. sporotrichioides* transformants were grown in YEPD for 7 days, while *Trichoderma* transformants were grown on PDB medium for only 48 h.

Further qPCR analysis revealed that expression of *tri5* and *tri4* was significantly increased in two of the three of the transformants analyzed, relative to expression in the untransformed *F. sporotrichioides* Δ*tri13*-mutant (Fig. 7).

**Fig. 8:**
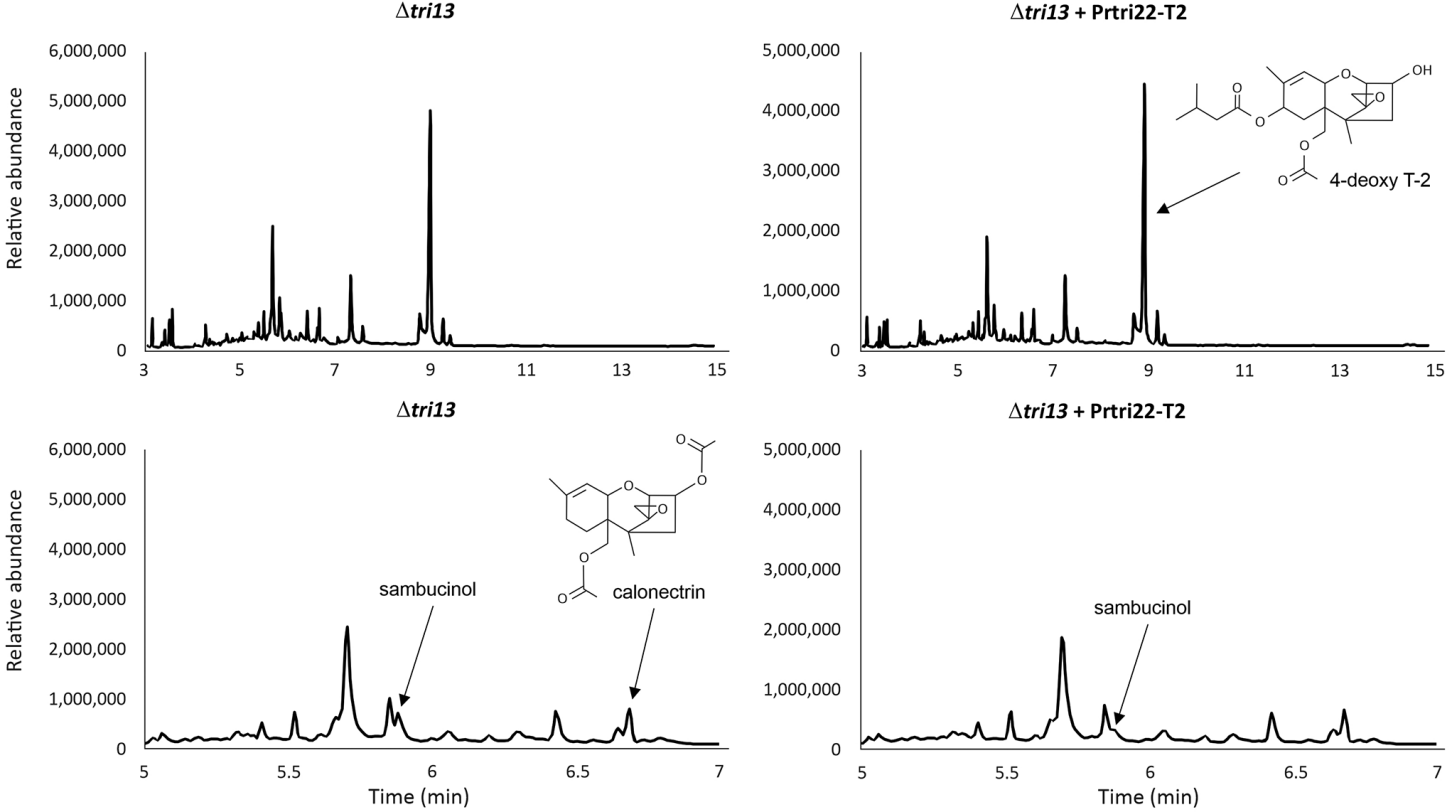
Gas chromatography-mass spectrometry analysis of extracts of cultures of the *F. sporotrichioides tri13* deletion mutant (Δ*tri13*, left panels) and the Δ*tri13* mutant transformed with *Paramyrothecium roridum tri22* expression construct, strain Δ*tri13* + Prtri22-T2 (= transformant T2) (right panels). Note the scale at the Y-axis could be different on each graphic and the scale fo the X-axis in the bottom panels was amplified for a more clear observation of some of the peaks important for this study

GC-MS analysis of culture extracts indicated that transformants T2, T8 and T9 had the same trichothecene production phenotype as the *F. sporotrichioides* Δ*tri13* mutant. That is, the Δ*tri13* mutant and transformants T2, T8 and T9 produced 4-deoxy T-2 toxin rather that T-2 toxin (Fig. 8). These results indicate that *P. roridum tri22* did not complement the *F. sporotrichioides* Δ*tri13* mutant (Fig. 8). To further confirm that *P. roridum TRI22* was functional in the *F. sporotrichioides* Δ*tri13* transformants, YEPD cultures of these transformants were fed EPT, the substrate of TRI22, and subsequently the cultures were examined by GC-MS for the presence of both EPT and trichodermol. The results require an indirect interpretation, because the levels of EPT in cultures of transformants T2, T8 and T9 decreased markedly but without evidence for conversion of EPT to trichodermol. Furthermore, feeding EPT to cultures of the *F. sporotrichioides* Δ*tri13* mutant resulted in production of sambucinol (Fig. S7, Fig. 8). Considering that sambucinol should be most likely produced from EPT (Zamir et al. 1999), the lack of conversion of EPT to sambucinol in transformants of Δ*tri13* mutant expressing *P. roridum tri22* indicates that the added EPT was likely processed to another metabolite. However, trichodermol, the product expected by C-4 hydroxylation of EPT, was not detected in the cultures, which could be due to a further modification of this compound, e.g., by a *Fusarium* glucosyltransferase. This hypothesis is consistent with previous results showing that when trichodermol was fed to *F. sporotrichioides* and *F. graminearum* cultures, it was transformed to trichodermol-4-*O*-α-glucopyranoside (Matsui et al. 2021).

### Secondary metabolites produced by *T. arundinaceum* strains

As noted above, the *T. arundinaceum* Δ*tri22* mutant produced only trace amounts of HA but relatively high levels of EPT, which was undetectable in cultures of the wild-type progenitor strain grown under the same conditions. GC-MS analysis indicated that the Δ*tri22* mutant also produced sambucinol and deoxysambucinol (Fig. 5), which previous studies indicate are likely formed by modification of EPT (Zamir et al. 1990, 1999). In addition, the GC-MS analysis revealed that the Δ*tri22* mutant produced aspinolides (Fig. 5). The latter finding is consistent with previous studies showing that loss of HA production in *T. arundinaceum* results in production of aspinolides (Cardoza et al. 2022a).

In contrast to the Δ*tri22* mutant, the complemented Δ*tri22* mutant carrying the *T. arundinaceum tri22* overexpression plasmid (i.e., transformants T6 and T9) produced no detectable EPT, sambucinol or deoxysambucinol. However, transformants T6 and T9 did produce trichodermol, which is formed by TRI22-catalyzed 4-hydroxylation of EPT. These results indicate that complementation of the *tri22* deletion restored the wild-type pattern of production of trichothecene intermediates. However, the levels of trichodermol produced by transformants T6 and T9 were lower than those produced by the wild-type progenitor strain. This is consistent with the lower levels of HA produced by transformants T6 and T9 relative to the wild type. In addition, transformants T6 and T9 produced relatively high levels of aspinolides and diverse fatty acids that were not detected in cultures of the wild type or the Δ*tri22* mutant.

Interestingly, in addition to EPT, GC-MC analysis of transformants T1 and T4 carrying the *F. graminearum tri11* and transformants T4 and T6 carrying *F. longipes tri11*, showed that three of these transformants produced significant amounts of 15-hydroxy EPT (Fig. 5). These transformants also produced relatively high levels of deoxysambucinol and aspinolides, but the levels produced by transformants carrying *F. longipes tri11* were lower than those produced by transformants carrying *F. graminearum tri11*. No fatty acids or trichodermol were detected in the analysis of transformants carrying *tri11* (Fig. 5).

### Effect of *tri22* gene deletion on the antifungal activity against *Rhizoctonia solani*

An antifungal assay against the fungal phytopathogen *Rhizoctonia solani* R43 was carried out to determine the effect of deletion of *tri22* on biocontrol activity of *T. arundinaceum*, and the gene overexpression experiments, conducted in the present study, in the antifungal activity of the different analyzed strains.

**Fig. 9:**
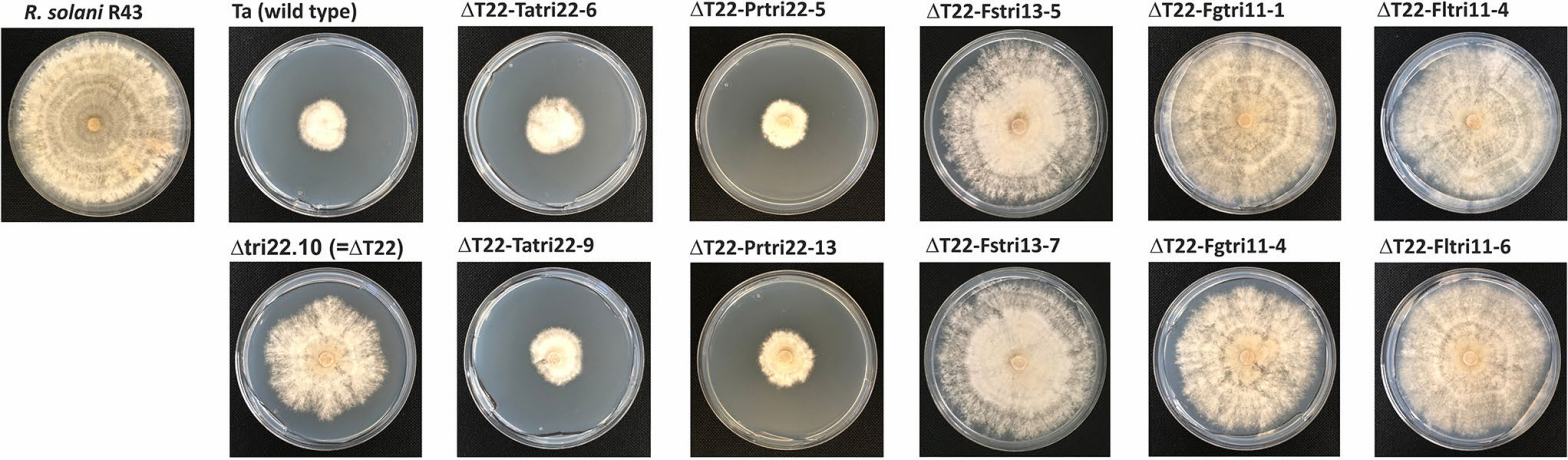
Antifungal activity of the *Trichoderma arundinaceum* strains generated in the current study against the fungal phytopathogen *Rhizoctonia solani* (strain R43) in a cellophane membrane assay. Strains used are described in the legend of Fig. 6. Ta- *T. arundinaceum*; Pr- *Paramyrothecium roridum*; Fs- *Fusarium sporotrichioides*; Fg- *F. graminearum*; Fl- *F. longipes*. ΔT22 = *T. arundinaceum* strain Dtri22.10 (i.e., Δ*tri22* mutant). Note: Each strain was assayed in triplicate (see Fig. S8)

*tri22*-gene deletion resulted in a remarkable reduction in the antifungal activity, which was estimated as a 50% of the radial growth inhibition (RI) activity observed for Ta37 wild-type strain. Transformation of Δ*tri22* mutant with plasmids designed for overexpression of *tri22* genes of *T. arundinaceum* and *P. roridum*, restored production of HA in the transformants but at lower levels than those found for in the wild-type strain. However, transformants overexpressing *P. roridum tri22* exhibit an even higher RI than the wild-type Ta37 strain (Fig. 9, Fig. S8, Table S2).

Transformants of the Δ*tri22* mutant expressing *F. sporotrichioides tri13, F. graminearum tri11, and F. longipes tri11* did not produce HA and, surprisingly, exhibited lower level of antifungal activity against *R. solani* than the Δ*tri22* mutant, with RI values significantly lower than those observed for the rest of Δ*tri22* transformants analyzed (Fig. 9, Fig. S8, Table S2). These results suggest a stimulatory effect of these transformants on *R. solani* growth.

### Effect of deoxysambucinol and sambucinol on *R. solani* growth

The rationale to perform this study was based in the results of the antifungal assays described above, where a stimulatory effect on growth of *R. solani* was observed when transformants of Δ*tri22* mutated strain overexpressing *F. longipes tri11*, *F. graminearum tri11*, and *F. sporotrichioides tri13*, were analyzed. As described above, these transformants produced significant amounts of two trichothecene-related compounds, i.e., deoxysambucinol and sambucinol, which were not detected in the wild-type strain, nor in the transformants overexpressing *T. arundinaceum tri22* gene. Thus, this assay was conducted to determine if the stimulatory effect of *R. solani* growth could be due to the production of these compounds.

Results indicate that these compounds, at concentrations from 0.0025 mg/mL to 10 mg/mL do not show any effect on *R. solani* growth at any of the concentrations assayed in comparison to control plates amended with methanol, which was the solvent used to prepare the deoxysambucinol and sambucinol solutions (Fig. S9).

## Discussion

Secondary metabolic enzymes tend to have relaxed substrate specificity, greater catalytic promiscuity, and lower activity compared to their counterparts in primary metabolism (Weng et al. 2012). Relaxed substrate specificity of trichothecene biosynthetic P450s and acetyltransferases in *Fusarium* is evident from the multiple trichothecene biosynthetic intermediates that individual enzymes can modify (Kimura et al. 2007; McCormick et al. 2011). The relaxed substrate specificity of the *Fusarium* acetyltransferase, TRI3, extends to trichodermol (Lindo et al. 2019), a trichothecene biosynthetic intermediate produced by *Trichoderma* species but not *Fusarium* species. In the current study, we used mutant complementation analysis to assess whether the substrate specificity of orthologs of three P450s (TRI11, TRI13 and TRI22) extends to trichothecene biosynthetic intermediates produced by fungi other than the genera in which the orthologs originated. Our results provide evidence that the three P450s exhibit more stringent substrate specificity than TRI3. Indeed, the only successful complementation occurred when *tri22* orthologs from *T. arundinaceum* or *P. roridum* were expressed in the *T. arundinaceum* Δ*tri22* mutant.

We hypothesized that the *F. sporotrichioides* TRI13 would compensate for the absence of TRI22 in the *T. arundinaceum* Δ*tri22* mutant because TRI13 and TRI22 both catalyze trichothecene 4-hydroxylation, albeit using different substrates (Brown et al. 2001). However, failure of *F. sporotrichioides tri13* to complement the Δ*tri22* mutant disproved the hypothesis. A reciprocal experiment in which we attempted to use *P. roridum tri22* to complement the *F. sporotrichioides* Δ*tri13* mutant also failed (Fig. 8). Together, these results suggest that TRI13 cannot utilize the TRI22 substrate produced by *T. arundinaceum* (i.e., EPT), and TRI22 cannot utilize TRI13 substrates produced by *F. sporotrichioides* (e.g., isotrichodermin = 3-acetyl EPT).

Although, *Fusarium* TRI11 orthologs catalyze trichothecene 15-hydroxylation, we hypothesized that they might also have trichothecene 4-hydroxylation activity based on the activity of *Fusarium* TRI3, a trichothecene acyltransferase that catalyzes acetylation of the 15-hydroxyl (15-*O*-acetylation) generated by TRI11. However, *Fusarium* TRI3 orthologs can also have low level of trichothecene 4-acetylation activity (Lindo et al. 2019; Tokai et al. 2008). Thus, we speculated that because TRI3 can modify trichothecenes at the C-4 position, albeit at low levels, the enzyme that TRI3 acts in tandem with (i.e., TRI11) might also have the ability to modify trichothecenes at the C-4 position. However, the finding that *F. graminearum tri11* and *F. longipes tri11* did not complement the *T. arundinaceum* Δ*tri22* mutant indicates that the flexibility of TRI11 does not extend to 4-hydroxylation of EPT. Furthermore, the ability of the Δ*tri22* mutant expressing *Fusarium tri11* orthologs to produce 15-hydroxy EPT indicates that TRI11 could have a broader substrate specificity than TRI13 and TRI22.

Cytochrome P450s play a critical role in biosynthesis and structural diversity of trichothecenes. For example, three P450 enzymes are essential for biosynthesis of HA: TRI4, TRI22 and TRI23. TRI4 catalyzes oxygenation of trichodiene at C2, C11, and C13 to produce isotrichodiol, which can spontaneously cyclize to form EPT (Fig. 1) (Cardoza et al. 2011); TRI22 catalyzes C-4 hydroxylation of EPT to form trichodermol; and TRI23 catalyzes one or more oxygenations required to convert octa-2,4,6-trieneoic to octa-2,4,6-trienedioic acid, the precursor of the C-4 substituent of HA (Cardoza et al. 2019) (Fig. 1). Prior to this study, the role of TRI22 in trichothecene biosynthesis was determined by feeding ETP to *Saccharomyces cerevisiae* that heterologously expressed the *T. arundinaceum tri22* homologs. In the current study, the trichothecene production phenotype of the *T. arundinaceum* Δ*tri22* mutant confirmed that the function of TRI22 was trichothecene 4-hydroxylation, which results in conversion of EPT to trichodermol (4-hydroxyl EPT). However, the *tri22* mutant also facilitated assessment of the effect of *tri22*-deletion on production of other trichothecene biosynthetic intermediates and other metabolites that were not detected in the wild-type *T. arundinaceum* strain.

The *T. arundinaceum* Δ*tri22* mutant produced high levels of EPT and very low levels of HA. Nevertheless, the Δ*tri22* mutant consistently produced low levels of HA that were 0.6% of the levels produced by the wild-type progenitor strain. An analogous leaky phenotype also resulted from deletion of the *T. arundinaceum tri3* gene, which encodes a trichothecene 4-*O-*acetyltransferase (Proctor et al. 2018). The *tri3* deletion (Δ*tri3*) mutant produced high levels of trichodermol (4-hydroxy EPT) as well as trace amounts of HA. The low levels of HA produced by the Δ*tri3* mutant were attributed to the activity of another nonspecific acetyl/acyltransferase that could catalyze the 4-*O-*acetylation reaction in an inefficient manner. Thus, the low level of HA produced by the *T. arundinaceum* Δ*tri22* mutant could result from the nonspecific activity of another oxygenase, possibly another P450 or a flavin-binding monooxygenase. In contrast to the leaky phenotypes resulting from deletion of *tri3* and *tri22*, deletion of other *tri* genes in *T. arundinaceum* (i.e., *tri5*, *tri17*, *tri18*, and *tri23*) did not result in production of trace levels of HA (Cardoza et al. 2019; Lindo et al. 2019; Proctor et al. 2018; Gutiérrez et al. 2021).

The evidence that complementation studies carried out by expression of *T. arundinaceum* and *P. roridum tri22* gene homologs were successful was that production of HA increased markedly in all transformants analyzed compared to production in the Δ*tri22* mutant (Table 1). However, the levels of HA produced in the complemented mutants was lower than the level detected for the wild-type strain. These low levels of HA production contrast the high level of expression of the *tri22* homologs in the complemented mutants. Thus, in the latter, expression of *tri22* was driven by the *Trichoderma harzianum tadir* promoter. Previous studies indicate that this promoter drives high levels of constitutive expression of *tri* genes (Cardoza et al. 2015, 2019; Lindo et al. 2019; Proctor et al. 2018). We speculate that the high level of constitutive expression of *tri22* in the current studies altered expression of other *tri* genes, which in turn reduced trichothecene production relative to the wild type. A similar reduction in HA production was observed in complementation experiments with Δ*tri17* and Δ*tri18* mutants of *T. arundinaceum* despite high levels of expression of *tri17* and *tri18* in the complemented mutants (Proctor et al. 2018; Lindo et al. 2019). Furthermore, successful complementation of Δ*tri22* mutant with both the *T. arundinaceum* and *P. roridum tri22* orthologs indicates that, despite the phylogenetic distance of these two species, the activity and substrate utilization of the corresponding TRI22 orthologs is conserved. This is consistent with the results from previous studies in which *P. roridum tri17* and *tri18* orthologs were used to complement the *T. arundinaceum* Δ*tri17* and Δ*tri18* mutants (Proctor et al. 2018; Lindo et al. 2019).

Lack of complementation of the *T. arundinaceum* Δ*tri22* mutant with the *Fusarium tri11* and *Fusarium tri13* orthologs as well as the lack of complementation of the *F. sporotrichioides* Δ*tri13* mutant with the *P. roridum tri22* ortholog are consistent with the distant relationships of TRI11, TRI13, and TRI22 that were revealed by phylogenetic analysis of predicted amino acid sequences of these enzymes and some of their closest relatives from trichothecene-producing and nonproducing fungi (Fig. 5). In the phylogenetic trees, TRI11, TRI22, and TRI13 are more distantly related to one another than each of them is to P450 enzymes involved in biochemical processes other than trichothecene biosynthesis.

Despite the almost total block in HA production observed in the Δ*tri22* mutants and in the transformants expressing *Fusarium tri11* and *tri13* genes, all these strains produced EPT, the substrate of TRI22, as well as several other secondary metabolites. Most notable was production of significant amounts of deoxysambucinol and sambucinol in cultures of the *tri22*-deleted mutant and in transformants of the mutant that expressed the *F. longipes* or *F. graminearum tri11* orthologs. The relationship of deoxysambucinol and sambucinol formation with accumulation of EPT was evident because these metabolites were not detected in transformants in which the *tri22* mutation was complemented (i.e., transformants of Δ*tri22* mutant with the *T. arundinaceum* and *P. roridum tri22*). In fact, production of deoxysambucinol and sambucinol has been previously described for *Fusarium* strains accumulating EPT, which was shown using radiolabeled intermediates that was incorporated into sambucinol via the intermediate deoxysambucinol (Zamir et al. 1990, 1996, 1999; Trapp et al. 1998). Accumulation of sambucinol was also shown for EPT producing *Trichoderma harzianum* transformants expressing *T. arundinaceum tri5* + *tri4* genes (Cardoza et al. 2015).

The *T. arundinaceum* Δ*tri22* mutant and transformants derived from it also produced aspinolides (Fig. 5). Such an increased production of aspinolides has been observed previously in other mutant strains of *T. arundinaceum* that were blocked in HA production (Izquierdo-Bueno et al. 2018; Malmierca et al. 2015; Cardoza et al. 2022a, b).

Deletion of *T. arundinaceum tri22* strongly affected the antifungal activity against *R. solani*, with a reduction near 50%. This activity was restored by complementation with *T. arundinaceum tri22* gene, and even in the case of complementation with *P. roridum tri22*, transformants caused greater inhibition of radial growth than the wild-type strain (Table S2). This result is noteworthy because HA production in the transformants was lower than in the wild-type strain (Table 1). As noted above, however, the transformants produced high levels of aspinolides than the wild-type strain. Because aspinolides have antifungal activity (Malmierca et al. 2015; Cardoza et al. 2022b), their increased production by transformants expressing *P. roridum tri22* might account for the increased antifungal activity of the transformants. Similar increases in antifungal activity and aspinolide production in transformants of *T. arundinaceum* have been observed previously (Malmierca et al. 2015; Cardoza et al. 2022b). In contrast to transformants expressing the *P. roridum tri22* ortholog, transformants expressing the *F. longipes tri11, F. graminearum tri11*, or *F. sporotrichioides tri13* stimulated *R. solani* growth (Fig. 9, Table S2). We hypothesized that deoxysambucinol and/or sambucinol stimulated *R. solani* growth. However, our finding that purified deoxysambucinol and sambucinol did not stimulate the growth of *R. solani* did not support this hypothesis. Thus, more studies are required to determine the cause of the stimulatory effect.

The results from the current study provide insights into variation in substrate specificity of trichothecene biosynthetic P450s. Results indicate that on one hand, TRI22 and TRI13 both catalyze trichothecene 4-hydroxylation, but that TRI22 cannot use the TRI13 substrate (3,15-diacetyl EPT), and TRI13 cannot use the TRI22 substrate (EPT). On the other hand, TRI11 can use the TRI22 substrate even though TRI11 and TRI22 catalyze hydroxylation of different positions of the core trichothecene structure. Thus, the results indicate that TRI13 and TRI22 have more stringent substrate specificities, while TRI11 has a less stringent substrate specificity. One factor that might affect this specificity is proximity of the position of the core trichothecene structure that enzyme modifies to a position with or without a substituent. This idea is consistent with the substrate specificities of TRI13 and TRI22 and the presence versus absence of a substituent at the position (C-3) adjacent to the position (C-4) that both enzymes hydroxylate. That is, TRI13 can hydroxylate 3,15-diacetyl EPT, which has a C-3 substituent, but it cannot hydroxylate EPT, which lacks a C-3 substituent. Conversely, TRI22 can hydroxylate EPT but cannot hydroxylate 3,15-diacetyl EPT. By contrast, the ability of TRI11 to hydroxylate C-15 of both EPT and 3-acetyl EPT, which differ in presence versus absence of a C-3 substituent, might reflect the distance of C-15 and C-3. A systematic analysis of the ability of TRI11, TRI13 and TRI22 to hydroxylate trichothecene analogs that vary in the presence, absence, and types of substituents at multiple positions of the core trichothecene structure should provide further insights into factors that affect substrate specificity of the enzymes. Such studies could also provide insight into factors that impact substrate specificity of trichothecene detoxification enzymes, which have potential to mitigate toxicity of animal feed contaminated with trichothecenes and to control crop diseases caused by trichothecene-producing *Fusarium* species (Desjardins et al. 1992, 1996; Proctor et al. 2018).

## Electronic supplementary material

Below is the link to the electronic supplementary material.

Supplementary Material 1

## Data Availability

The manuscript has no associated data.

## Declarations

### Disclaimer

Mention of trade names or commercial products in this publication is solely for the purpose of providing specific information and does not imply recommendation or endorsement by the U.S. Department of Agriculture. USDA is an equal opportunity provider and employer.
