# Supplementary material for: Analysis of substrate specificity of cytochrome P450 monooxygenases involved in trichothecene toxin biosynthesis
Source: Appl Microbiol Biotechnol. 2024 Jan 6;108:152. doi: 10.1007/s00253-023-12950-1 (PMC10771604; doi:10.1007/s00253-023-12950-1)
Supplement: Supplementary file 1 [file 253_2023_12950_MOESM1_ESM.pdf]

Supplementary Material

Journal: Applied Microbiology and Biotechnology

Analysis of substrate specificity of cytochrome P450 monooxygenases involved in trichothecene toxin biosynthesis

Rosa E. Cardoza[1#], Susan P. McCormick[2#], Natalia Martínez-Reyes[1], Joaquín Rodríguez-Fernández[3], Mark Busman[2], Robert H. Proctor[2]*, Santiago Gutiérrez[1]*

[1]University Group for Research in Engineering and Sustainable Agriculture (GUIIAS), Area of Microbiology, University of León, 24400 Ponferrada, Spain

[2]USDA, Agricultural Research Service, National Center for Agricultural Utilization Research, Mycotoxin Prevention and Applied Microbiology Research Unit, 1815 N University St., Peoria, Illinois, United States 61604

[3]Area of Biochemistry and Molecular Biology, University of León, 24400 Ponferrada, Spain.

#These authors contributed equally to this work

Corresponding authors*:

Santiago Gutiérrez (s.gutierrez@unileon.es), phone number: +34 987442060.

Robert H. Proctor (robert.proctor@ars.usda.gov), phone number: +1 309 6816380.

Table S1. Oligonucleotides used in the present work.

**Name**		**Sequence 5´-3´**	
**Construction of pΔtri22 to delete *tri22* gene.**			
TRI22_5r_F_BamHI (1,013 bp)		ggatccATGAGGTGGCCATTGGTT	
TRI22_5r_R_SmaI		cccgggTGTGATGAGATGCAGAGC	
TRI22_3r_F_SmaI (1,048 bp)		cccgggGACGAATTGGAACCCAGT	
TRI22_3r_R_SalI		gtcgacGTGCAGCACCGTGATAAT	
**Oligonucleotides to amplify an internal fragment to *tri22* gene.**			
T22_N (1,054 bp)		CGCAAGTATGGCAATGCA	
T22_C		GCTAATTGGTGGACAGAG	
**Amplification of 1,377 bp and 2,069 bp fragments corresponding to the 5´ and 3´ extremes, respectively, of the recombination cassettes designed for *tri22* gene deletion**			
Tri22-5r_ff (1,377 bp)		CGTCGTCCGTGATTCTAA	
TtrpC-d		GTAACCATGCATGGTTGC	
PgpdA-d (2,069 bp)		ATCATCCACTGCACCTCA	
Tri22-3r_rr		TCTCACGAGAAGGCATCT	
**Amplification of *tri22/TRI22/TRI11/TRI13* genes from *Trichoderma* and non-*Trichoderma* species**			
Trichoderma arundinaceum	TARUN_T22_ATG (1,580 bp)	GCCAACGCTATTTCGGTG	
	TARUN_T22_end	TCATTTACTGGCGAATCG	
Paramyrothecium roridum	PRORI_T22_ATG (1,558 bp)	TATGCCCTTTTGGTCAAG	
	PRORI_T22_end	TTAAAAGACACGGTTCTT	
Fusarium graminearum	FGRAM_T11_ATG (1,741 bp)	TTTCAATACTCCCTGTGG	
	FGRAM_T11_end	TCACCTTGGGTCAAGGTA	
Fusarium longipes	FLONG_T11_ATG (1,715 bp)	ATGGAACACACTATCTGG	
	FLONG_T11_end	TCACCTTGGATTAAGGTA	
Fusarium sporotrichioides	FSPOR_T13_ATG (1,833 bp)	TTTCTCTCTCTCTGTCTA	
	FSPOR_T13_end	CTATGTCGTTGTTAAATC	
**Oligos for qPCR analysis**			
			Efficiency
T. arundinaceum α-actin	aactinF	ACTGGGACGACATGGAGAAG	92.1%
	aactinR	GGCCTGGATGGAGACATAGA	
T. arundinaceum tri22	tri22-2F	TTCCCATCCTGACAACTTCC	99.6%
	tri22-2R	ATGGCGAAGAGGGGTTAGTT	
F. graminearum TRI11	FG_T11_L1	ATGAGCAGTCATCCAGTGGT	97.7%
	FG_T11_R1	CTCTCAAAGGCCAGAGCAAC	
F. longipes TRI11	FL_T11_L1	CCAAGATTGCTCCCTTGCTC	85.7%
	FL_T11_R1	CGTGATCTTGCCCACGAAAT	
P. roridum TRI22	PR_T22_L1	TGCTTGAGCTCTTCACTCCA	92.7%
	PR_T22_R1	TCGTTGAGACCTTGAGGCTT	
F. sporotrichioides TRI13	FSPOR_T13_L1	ACTCGTGCGAGTATGGGATT	108%
	FSPOR_T13_R1	AACTCCCGAAAGCAAAGCTG	
F. sporotrichioides TRI5	FSPOR_T5_L1	CTCTGCACTCGTCCAAACAG	109%
	FSPOR_T5_R1	TGAGCGTCCTCAGTCTTCTC	
F. sporotrichioides TRI4	FSPOR_T4_L1	GCCGATGTCATCACTTCTCG	102%
	FSPOR_T4_R1	GATGAAGCGTGCAAGATGGT	
**Oligos for checking overexpressing transformants**			
pTC_TARUN_T22_ble_b<br>pTC_PRORI_T22_ble_b<br>pTC_FGRAM_T11_ble_b<br>pTC_FLONG_T11_ble_a	Pta	CACTGCAGTCCACATTGA	
	Tcbh2	CTGGCAACAATCCATCAG	

pTC_FSPOR_TRI13a_ble_a<br>pTC_PRORI_T22a		

Table S2. Percentages of radial growth inhibition (RI) of *R. solani* by strains analyzed in the present work after 7 days of incubation after pathogen´s plug disposal.

		**% RI/% RI in Ta37***
**Ta37**	63.48^{a} ± 7.94	100
**Δtri22.10 (=ΔT22)**	31.80^{b} ± 5.11	50.09
**ΔT22-Tatri22-6**	59.72^{a} ± 7.56	94.07
**ΔT22-Tatri22-9**	63.09^{a} ± 2.35	99.36
**ΔT22-Prtri22-5**	74.52^{c} ± 2.97	117.39
**ΔT22-Prtri22-13**	73.59^{a,c} ± 6.81	115.92
**ΔT22-Fstri13-5**	2.83^{e} ± 4.30	0.04
**ΔT22-Fstri13-7**	1.05^{e} ± 2.61	0.02
**ΔT22-Fgtri11-1**	-0.23^{d} ± 1.71	0.00
**ΔT22-Fgtri11-4**	11.31^{e} ± 2.11	17.81
**ΔT22-Fltri11-4**	16.23^{e} ± 4.72	25.56
**ΔT22-Fltri11-6**	2.17^{e} ± 3.02	0.03

n= 3, ANOVA.

[a,b,c,d,e] On each column, values followed by different superscript letters are significantly different ($p < 0.05$).

*This column includes the ratio of radial growth inhibition (RI) for each strain versus that for the wild-type strain, assigning a ratio of 100% to the values observed for the wild-type strain.

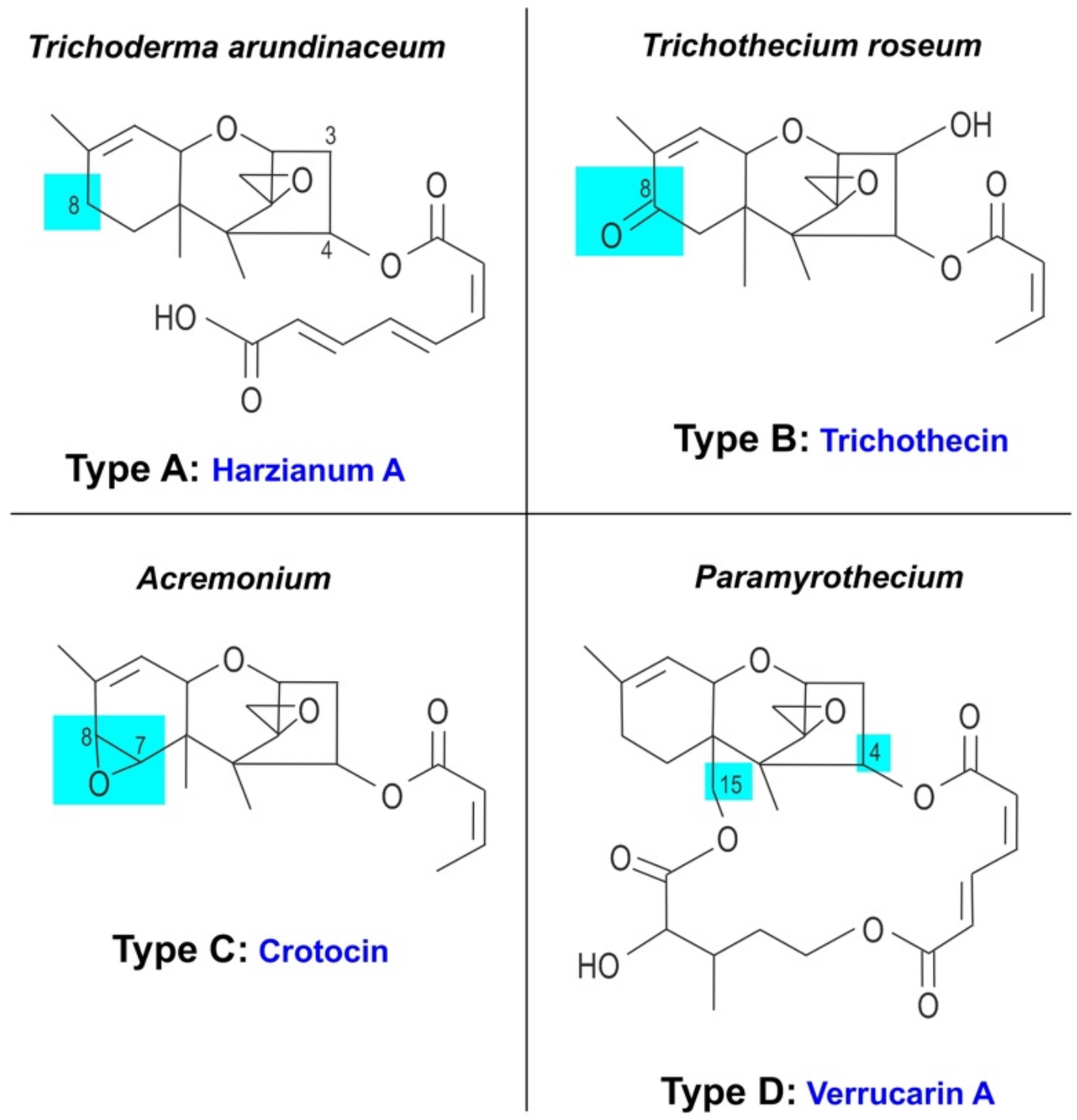

Fig. S1. Examples of chemical structures of type A, B, C and D trichothecenes. The positions and/or functional groups upon which the classification system is based are highlighted with blue shading.

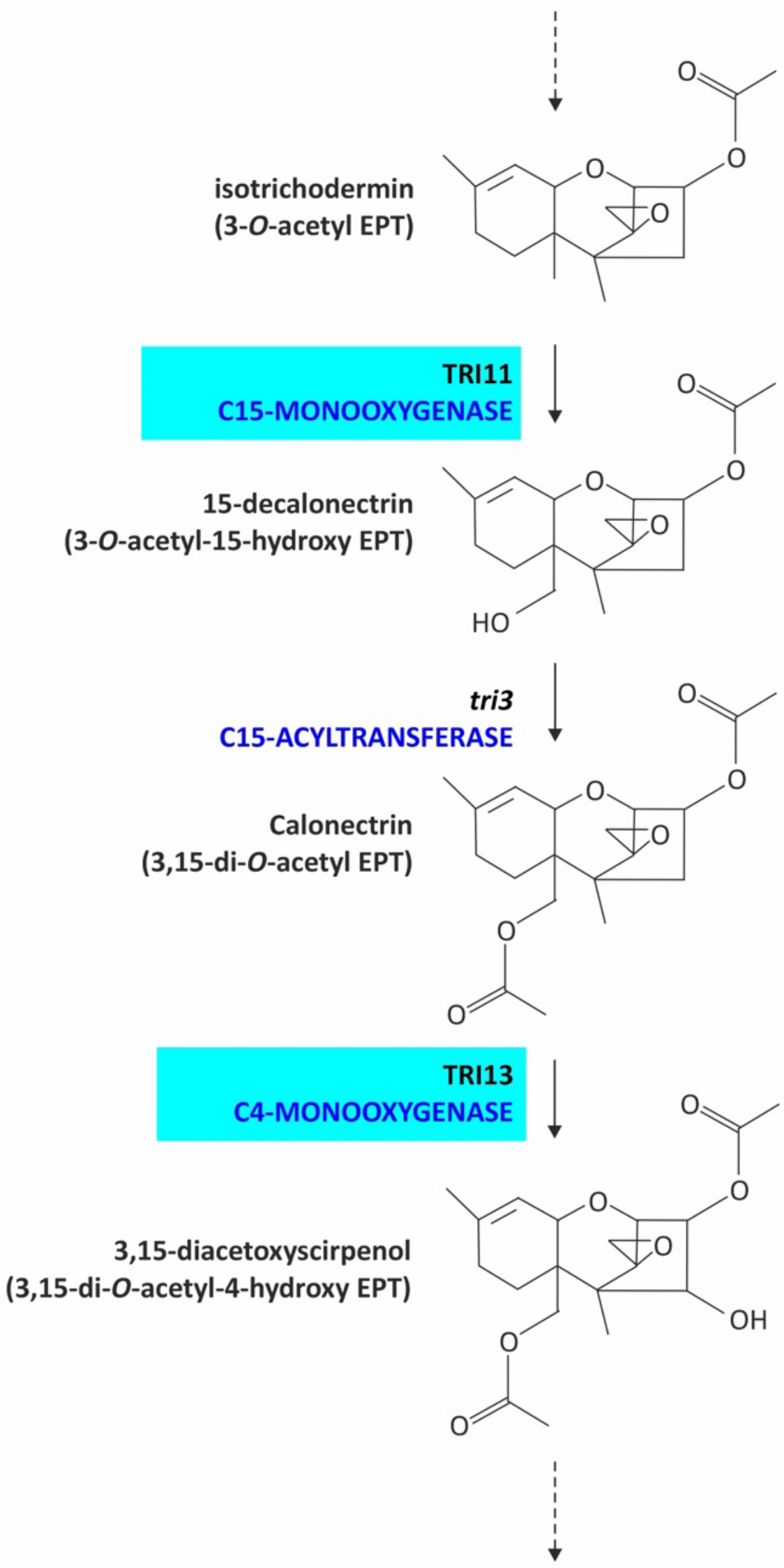

Fig. S2. Schematic pathway showing the step in the biosynthesis of *Fusarium* trichothecenes catalyzed by TRI11 and TRI13, two cytochrome P450 monooxygenases that have been analyzed in the present work (blue shaded). Enzymes and enzymatic activities are written in uppercase black and blue letters, respecitvely.

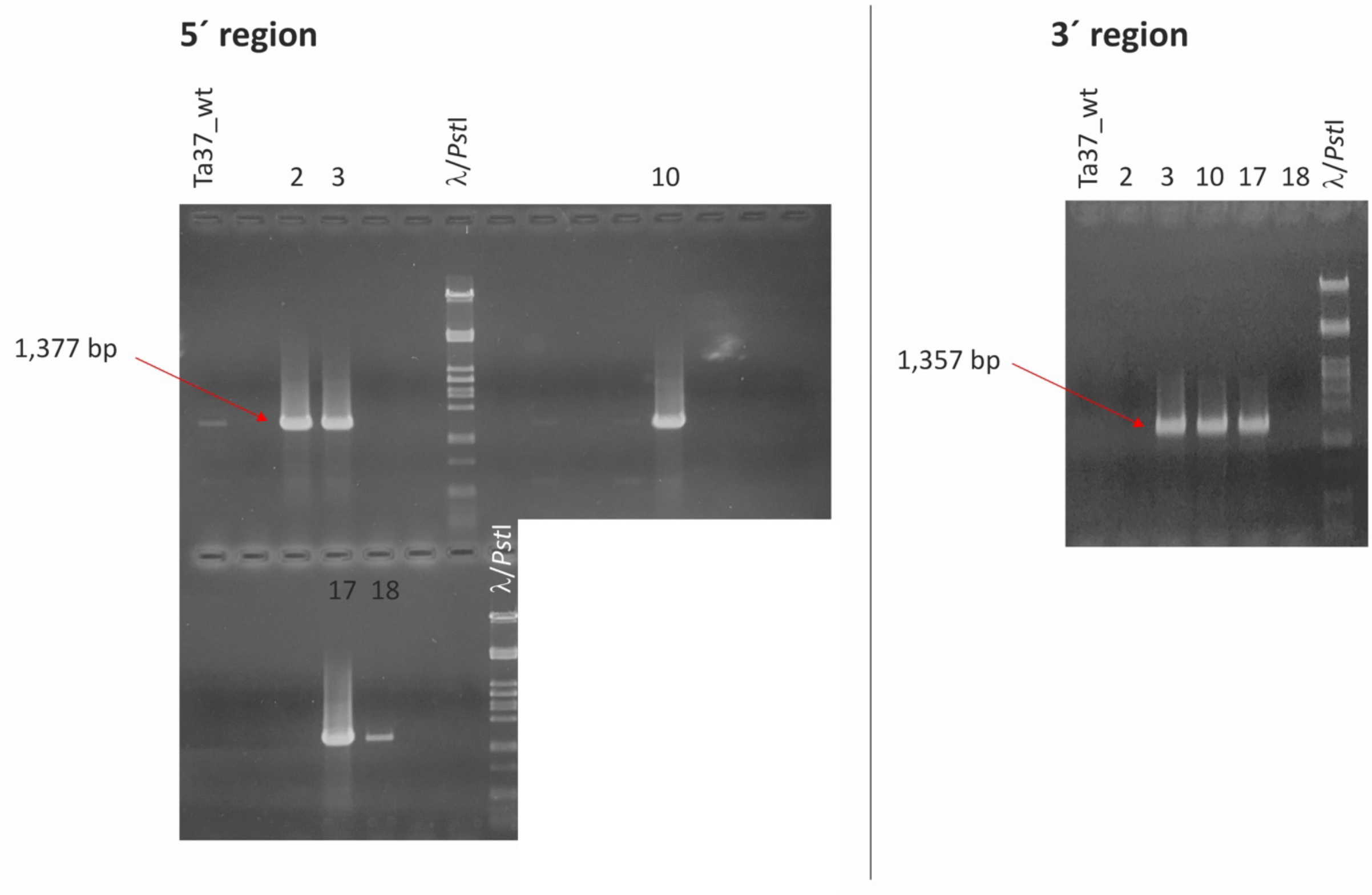

Fig. S3. PCR analysis of selected *T. arundinaceum* transfomants obtained with plasmid pΔ*tri22* using primer pairs tri22-5rr/TtrpC-d (**left panel**), and PgpdA-d/tri22-3rr (**right panel**) that amplify 1,377 bp and 1,357 DNA fragments corresponding to the 5´ and 3´ extremes of the recombination cassette designed for *tri22* deletion. λ/*PstI* = DNA from phage lambda digested with *PstI*, used as molecular size marker. Transformants #3, #10, and #17 exhibited the expected PCR pattern, which indicates replacement of *tri22* with *hph*.

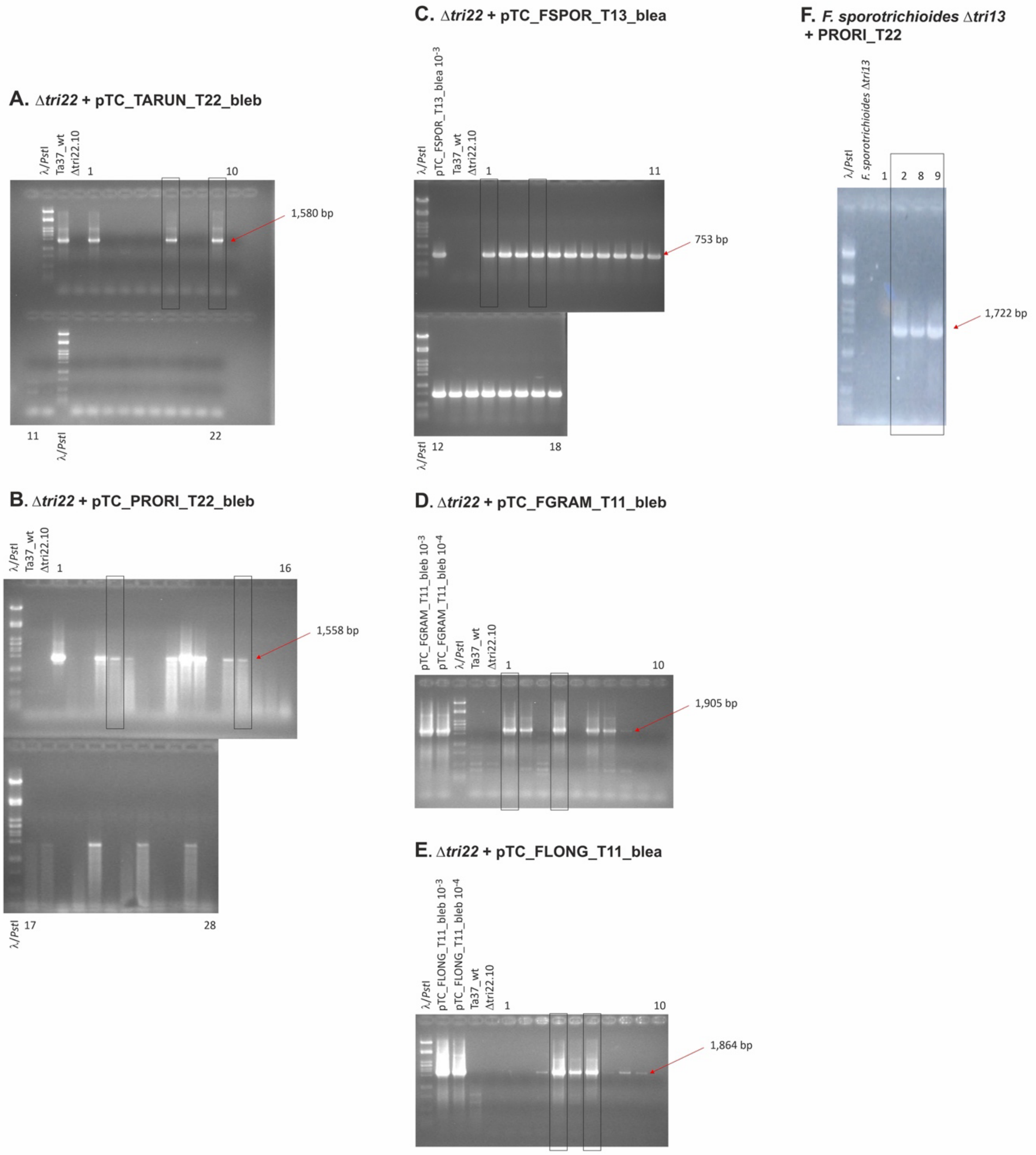

Fig. S4. Agarose gel electrophoresis of PCR reactions carry out to detect transformants of Δ*tri22* mutant that have incorporated the different constructs designed for overexpression of: **A**. *T. arundinaceum tri22*; **B**. *P. roridum tri22*; **C**. *F. sporotrichioides tri13*; **D**. *F. graminearum tri11*; and **E**. *F. longipes tri11*. **F**. gel electrophoresis of PCR reactions to detect transformants of *F. sporotrichioides* Δ*tri13* mutant that have incorporated *P. roridum tri22* gene.
Size of the expected fragments for each PCR reaction are shown at the right of each agarose gel. Transformants selected on each experiment were squared with a black lined rectangle. Oligonucleotide pairs used in the different analyses are indicated in the text. λ/*Pst*I, lambda phage DNA digested with the endonuclease *Pst*I, used as a DNA size marker.

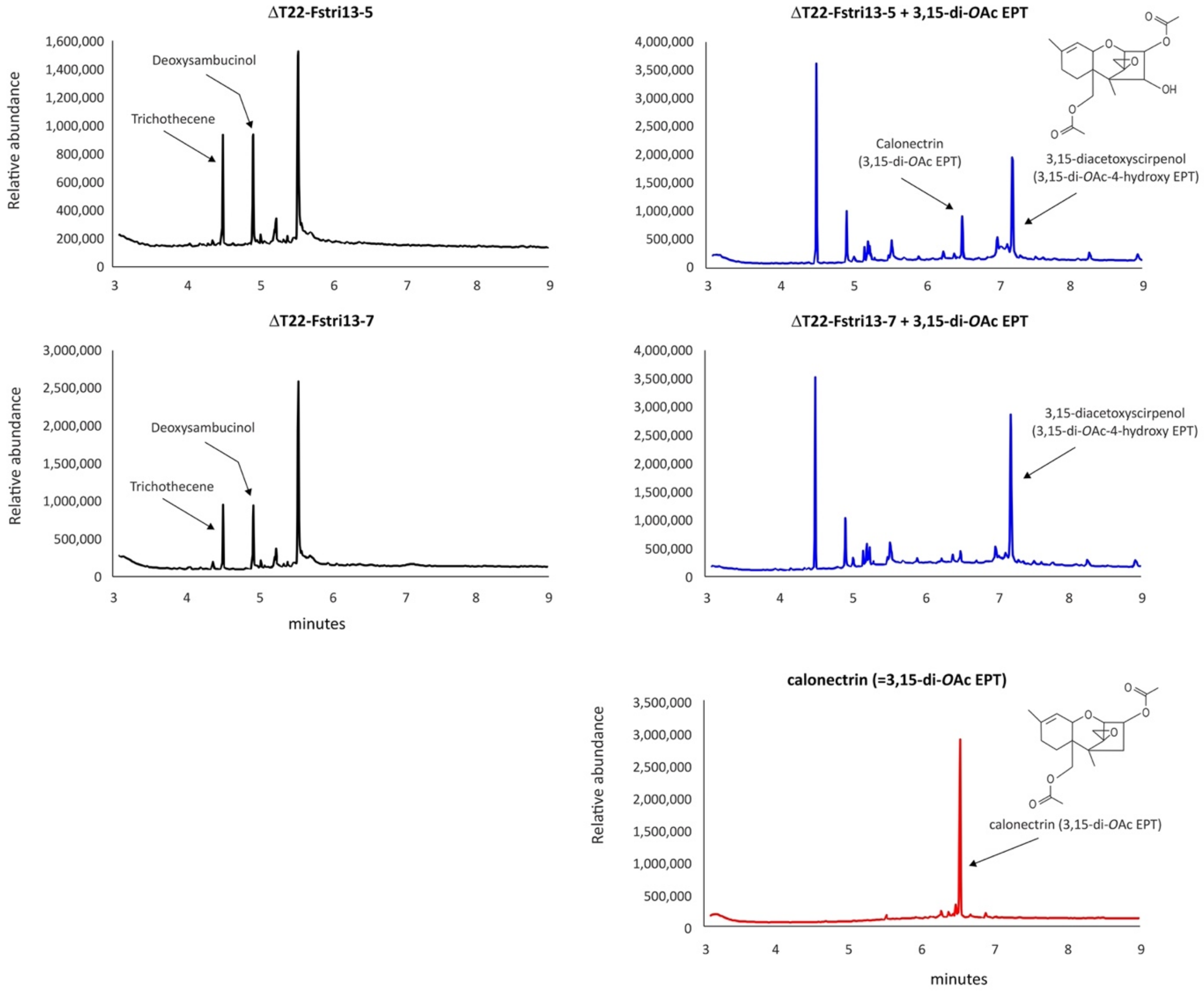

Fig. S5. Reconstructed ion chromatograms of extracts from 7-days old YEPD cultures of ΔT22-Fstri13-5 and ΔT22-Fstri13-7 transformants before (left panels), and after feeding calonectrin (right panels). Left bottom panel corresponds to a reconstructed ion chromatogram of pure calonectrin used in the feeding experiments.

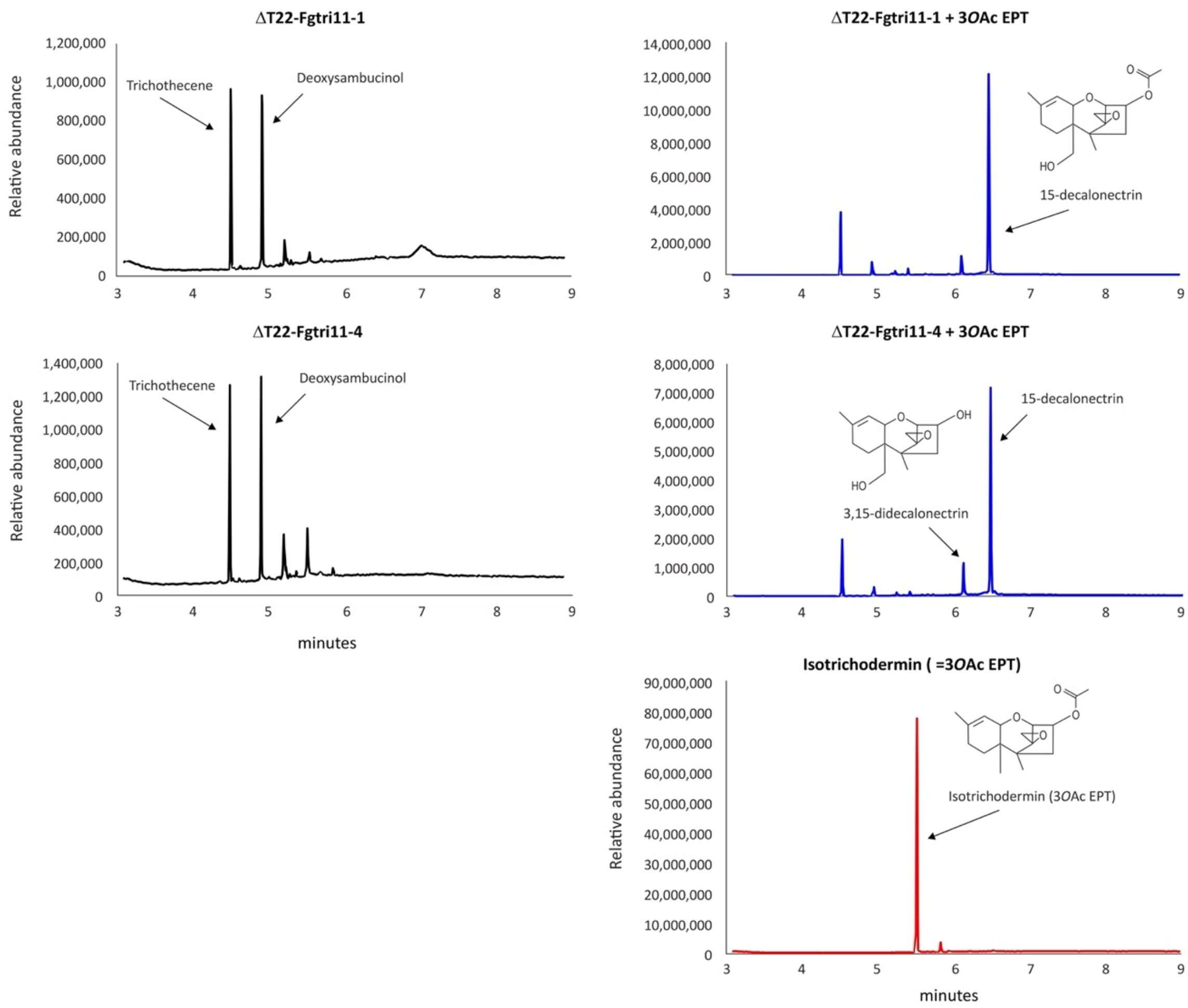

Fig. S6. Reconstructed ion chromatograms of extracts from 7-days old YEPD cultures of ΔT22-Fgtri11-1 and ΔT22-Fgtri11-4 transformants before (left panels), and after feeding isotrichodermin (right panels). Left bottom panel corresponds to a reconstructed ion chromatogram of pure isotrichodermin used in the feeding experiments.

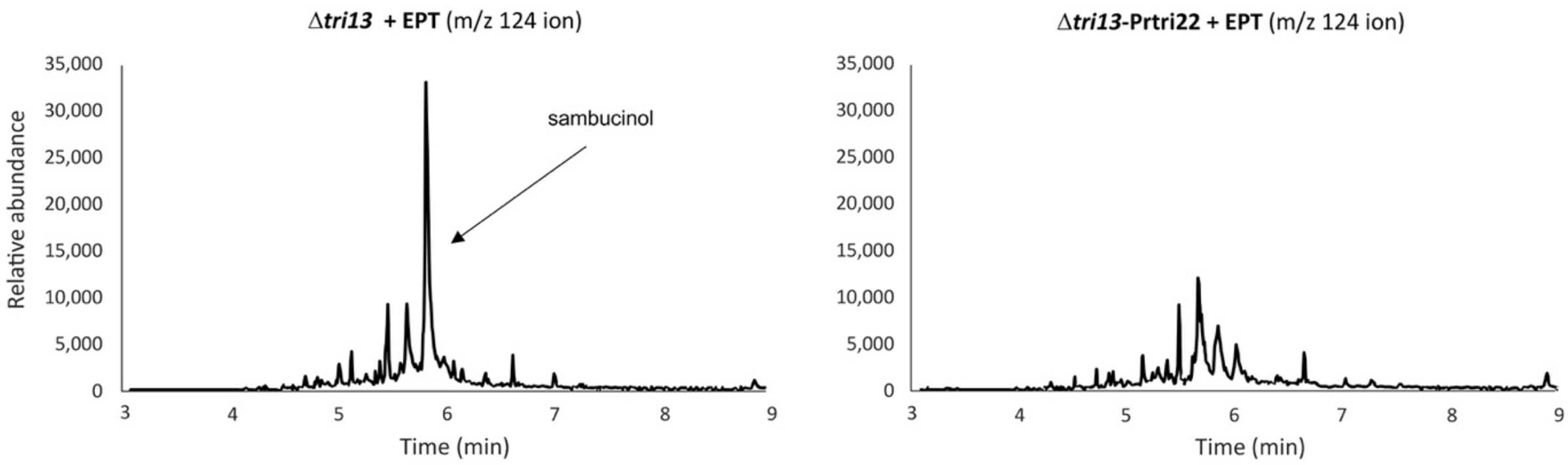

Fig. S7. Differential mass spectra for m/z 124 ion, which specifically picks out sambucinol, observed from broths of DFsTRI13 (Δ*tri13*) mutant (left panel) and transformants of that strain expressing *Paramyrothecium roridum tri22* gene (Prtri22) (right panel), in both cases amended with EPT. Note that sambucinol was detected at much higher level in the mutant without *P. roridum tri22.*

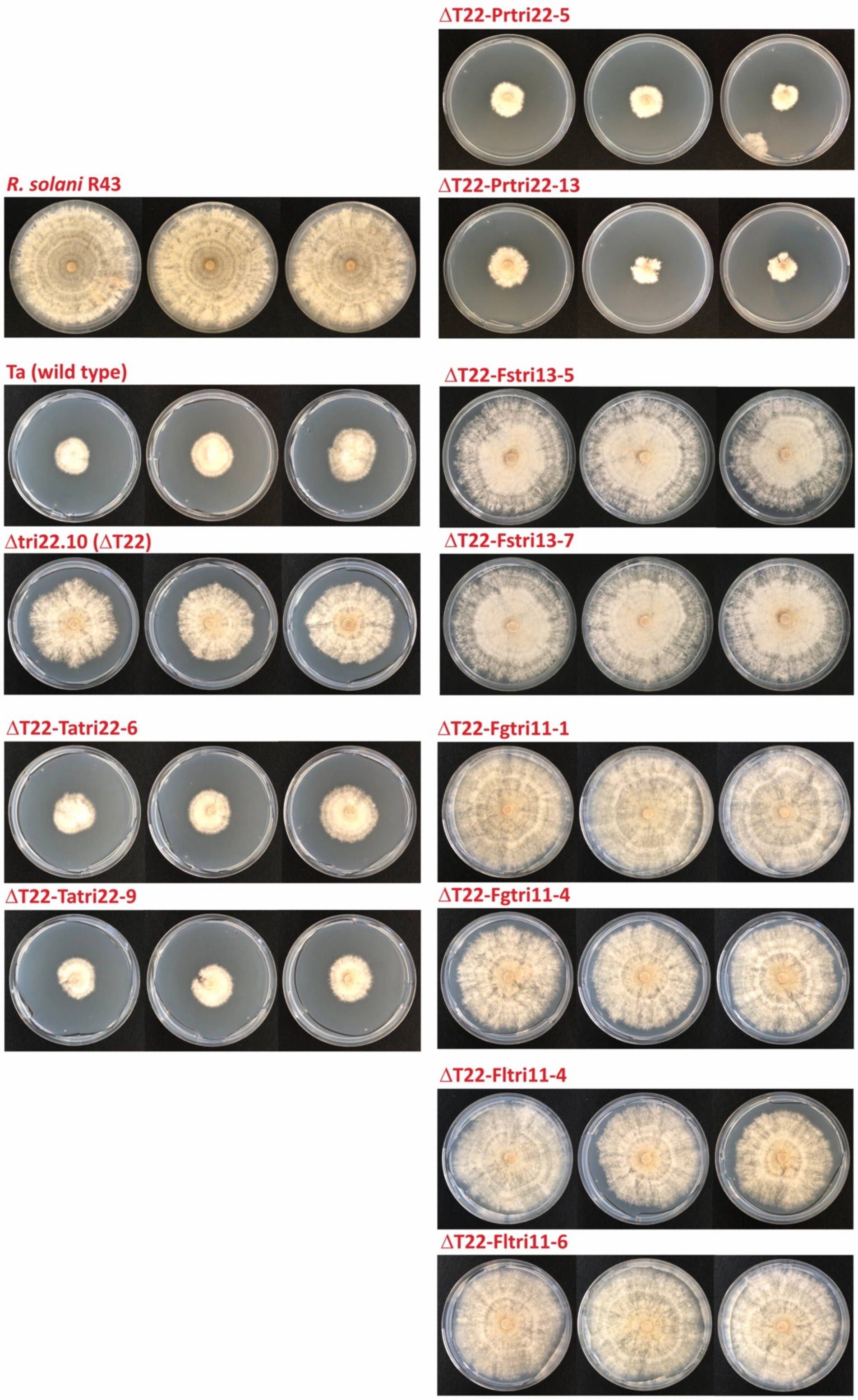

Fig. S8. Antifungal activity of the *T. arundinaceum* strains analyzed in the current study against the fungal phytopathogen *Rhizoctonia solani* (strain R43) in a cellophane membrane assay. Strains used were described in the legend to **Fig. 6**. Ta- *Trichoderma arundinaceum*; Pr- *Paramyrothecium roridum*; Fs- *F. sporotrichioides*; Fg- *Fusarium graminearum*; Fl- *F. longipes*. ΔT22= *T. arundinaceum* Δtri22.10 mutant.

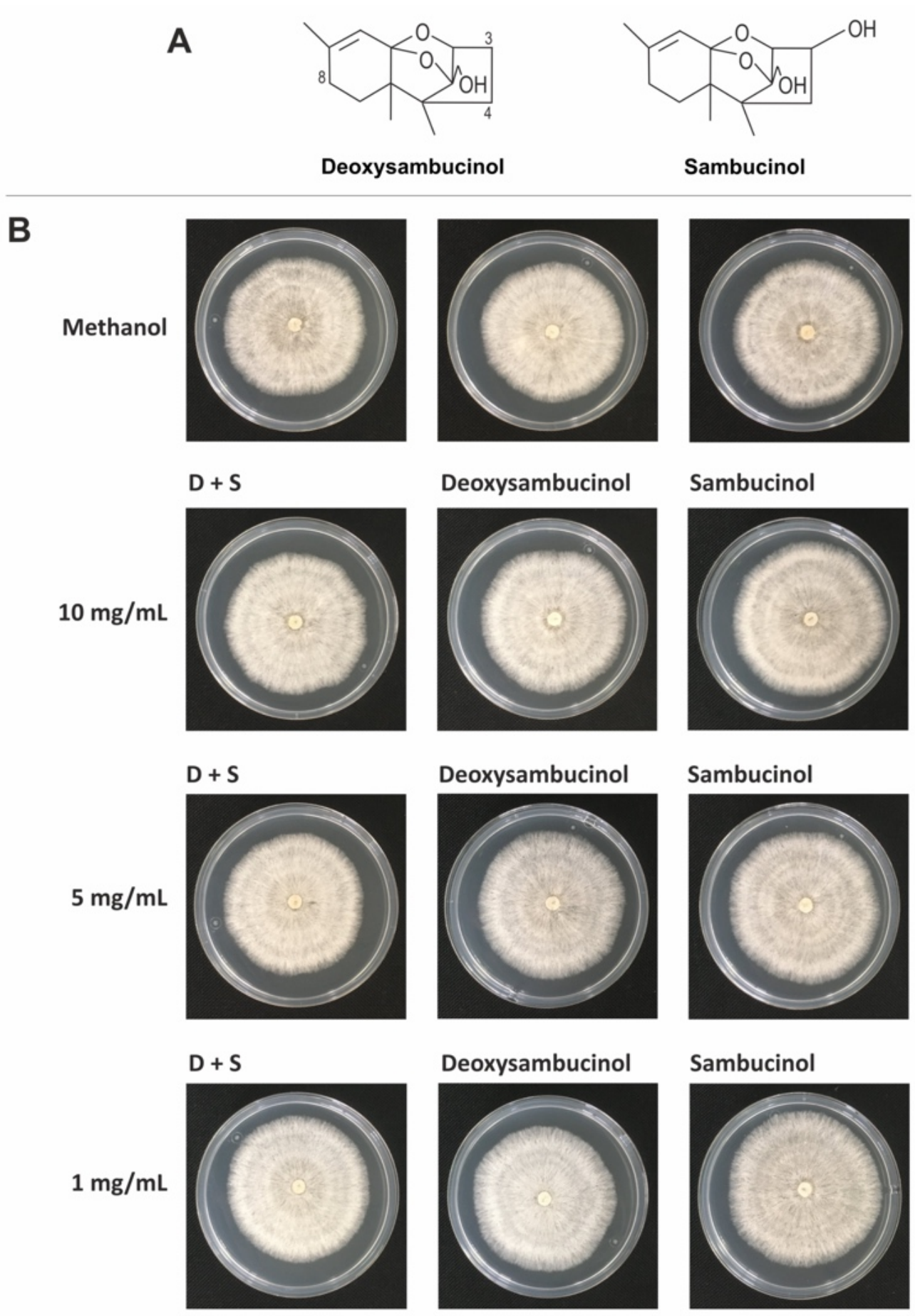

Fig. S9. **A**. Chemical structure of deoxysambucinol and sambucinol. **B**. Effect of deoxysambucinol and sambucinol (1 mg/mL, 5 mg/mL, and 10 mg/mL) on *R. solani* growth. Plates labeled as D + S were amended with both compounds at the final concentration indicated at the left.

Supplementary File 1. Amino acid sequences used for the phylogenetic study illustrated in **Fig. 3**.

>*Aspergillus alliaceus* CBS 536.65 TRI22
MISTTTLVGGVVGVYLLLSLSRTIYNLFFHPLAYLPGPKLWVAFPVFRQVSSIRGIFDARMKEFHQVYGDVVRFGPN
EVSFITEKAWRDIYDHRPNQLERFILSTTRRPDIFDADEVNHARYRKALNPAFSLKGLQDQEPVVKGYIDQFISRLKD
EAKTGAPTDMVKWYNFTTFDIIGDLAFGESFGGLRNKEYHFTISFTFEAFKLLSYLEAGAAYPLLLKLLMLFTPKSLVE
ARDRKEEHARVTVNKRLNNKALHGRGDFMDAMLRNRGEKQGLTDPELVANASTLITAGSETTATVLSGITYFLLRN
PEKLKKVTDEVRSTFNSEDDIVFITASSRLPYMIACFQEALRLYPPVPTGMPRVTPEAGLTEISGHNIPPNTKVSVHQL
AAYYHPKNFNQPEKYIPERWLPEAKTDPSSPYYNDAREVLQPFNVGPRNCIGRNLAYSEMRVMLARVLWNFDLEL
SPASENWNQQKSHFLWEKSGLMCVLKDRFK*
>*Aspergillus coremiiformis* CBS 553.77 TRI22
MPSVIATVGSIVGVYVLFALSRIVYNVFFHPLAYLPGPKLWIALPICRQIASIRGVFDARMRDLHQRYGEVIRFAPDEV
SFITEQAWRDIYDHRPNQLQRFILSTTRRPDIFDSNEADHARIRKVLNPAFSPKGLQAQEPLVKGYVDQFIDQLRDE
AKTGAPTNMVQWYNFTTFDIIGDLAFGESFGGLRNKAYHFTISFTFEAFKLLTYLEAGAAYPLLLKALMLFTPTSLIEA
RDRKEQHARETVEKRLHNEALHGRGDFMDAMLRHRGEKQGLTDPELVANASTLITAGSETTATILSGITYFLLRNP
DKLDKLTQEVRGAFDAEDDIVFSTATARLPYMIACFQEAFRLYPPVPTGMPRMTPESGWTEISGHQIPPNIKVSVH
QLAAYYHPKNFAHPEQYAPERWLPEARTDAASPFYHDAREALQPFNVGPRNCIGRHLAYNEMRVILARVLWNFD
LALSPESEHWTQQQSYFLWDKPGLMCVLKDRFE*
>*Aspergillus hancockii* TRI22
MISTGVIVGGLVGFYVLLSLARTFYNLYFHPLAYLPGPKLWIAFPVFRQITSIRGLLDTRMREFHLTYGDVIRFGPDEV
SFITEKAWRDIYDHRPNQLERFILSTTRRPDIFDANEVDHNRFRKALNPAFSPKGLQDQEPVVKGYIDTFISRLRDEA
KVGAPTDMVKWYNFTTFDIIGDLAFGESFGGLKNKEYHFTISFTFEAFKLLTYLEAGHAYPLLLKLLMLFTPKSLIEAR
DMKEQHAADTVNKRLNNKALHGRGDFMDAMLRNRGEKQGLNDAELVANASTLITAGSETTATILSGITYFLLKNP
DKLKKVTEEVRSAFDSEDDIVFNTASSRLPYMIAAFQEAFRLYPPVPSGLQRMTPTSGTTDISGHNIPPNTKVSVHQL
AAYYHPNNFKKPEEYIPERWLPEARSDPSSPYYNDSREVLQPFNVGPRNCIGRNLAYNEMRVMLARVLYNFDLELA
PESENWNQQKSHFLWEKPGLMCVLKDRFK*
>*Aspergillus leporis* CBS 151.66 TRI22
MASTGVLVGGLVGFYVLVSFARTIYNLFFHPLAYLPGPKLWVAFPVFRQVTSIRGLFDARMREFHQVYGEVIRFGP
DEVSFITEKAWRDIYDHRPNQLERFILSSTRRPDIFDANEVDHNRYRKALNPAFSPKGLQDQEPVVKGYLDMFISRL
RDEAKIGAPTDMVKWYNFTTFDIIGDLAFGESFGGLRNKEYHFTISFTFEAFKLLSYLEAGHAYPLLLKLLMLFTPKSLI
EARDRKEKHAADTVNKRLNNKALHGRGDFMDAMLRNRGEKQGLNDAELVANASTLITAGSETTATVLSGITYFLL
RNPDKLKRVTDEVRSSFESEDDIVFTTASSRLPYMIACFQEALRLYPPVPTGMPRMTPTSGLTDISGHNIPPNTKVSV
HQLAAYYHPKNFHQPEKYVPERWLPETREDSSSPYYNDSREVLQPFNVGPRNCIGRNLAYNEMRVMLARVLYNF
DLELSPESEHWNQQKSHFLWEKPRLMCVLKDRFK*
>*Beauveria bassiana* ARSEF 2860 TRI22
MPADIIGIAKVLMAIYSVSFVGTILYRLYLHPLSNFSGPKSWIAFPLLRQIAHIRGLLDARMCDFHRIYGPVVRFGPGE
VSFITEDAWRDIYDHKPNQLQRFILPTARRPDIFDAEEADHDRYRKAMSHAFSPRGLLQQEPVVNGYIDMLMGRL
HEQAARGASVDMTMWYTLTLFDVIGDLAFGQSFGGLRDQVLHSTISFTFEAFKLLTFLEAGASFPILFKLLQLCIPKR
LIEARDRKEKHAEETVRRRMADESLHGRGDFMDSMMRHDGTPQGLNTRELIANASTLITAGSETTSTILSGVTFYLL
CNIDAMKKVVNEVRSAYSSESEIAMSTTAQRLPYMSACFLEAFRLYPPVPSGLQRMVPSEGRTRVSSYDIPPNTKVS
VHPLAAYNDARNWHKPELFLPERWLPEAKSDPSSLFYNDCRNVCQPFSVGPRSCPGRNMAEQEYRLILARILWNF
DLELCPESKNWTEQRTHYLWEKKPLMCRLNPRPAVA*
>*Beauveria bassiana* ARSEF 2860 TRI11
MSPVLAIAARLITRKIIVMTDDSYLRCATDALTQPSIWNITLLVLIVTFIYAVFKTVYNVFLHPLRKFPGPWLYTLTSIPH
TRLTLSGLSHKKHLELHLKYGPVVRIAPNMLSFNHPDAMKDLRGHRKTGEPEHGKDPISIPFNLDNIVGSNREHHTR
FRRALAHGFSSQAMLEQEPTFKAYVDQLLGRLEENSSDGTVAVDIASWYTFTAFDLIGDLAFGESFGCLDNSNYHP
WVALTFQSLKSLAFNTEIGRYPSIAMLVQLLPRGIFSKFAENKKLSSAKVRKRLDSGSNRPDFIGKITEGSRSKPTEINF
EELASNASVLIVAGSETTATLLSAAIYFLTKNPQAFAMLAEEIRGAFTNKDEIGLITTQGLPYMQAVLDEALRLYPPVA
GGGSPRKVAKGGVQIAGYFVPENTLVENDMWALHHNPKYFAQPESFVPERWLGHKDFQDDQLGAVKPFSIGPR
NCIGMKYVLALDIHDTPEL*
>*Cordyceps confragosa* RCEF 1005 TRI22

MAADLINIVKVLVSIYLVSSVGRILYSLYLHPLSKFPGPNIWIAFPLLRQIAHIRGLLDARLCDFHRTYGPVVRFGPGEV
SFITEDAWRDIYDHKPNQLQRFILPTARRPDIFDSNEADHDRYRKAMSHSFSPRGLMQQEPVVKGYIDMLMGRLH
EQAARGASVDMTMWYTLTLFDVIGDLAFGQSFGGLRDQVIHSTISFTFEAFKLLTYLEAGASFPLLLKLLQLWTPKR
LIEARDRKEAHAEETVRRRMADKSLHGRGDFLDSMMRYEGTPQSLNEKELIANASTLIVAGSETTSTILSGLTFYLLR
NPDAYKKVADEVRSAYAAEAEIVMSTTAQRLPYMSACFQEAFRLYPPVPSGLQRVAPPDGKTIVAGHEIPPNTKVF
VHPLAAYTDPSNWHKPDSFSPERWLPDAKSDPSSPFYKDCRNVCQPFSVGPRSCPGRNMAEQEYRLIMARILWN
FDLQLCLESKDWTQQRTHYLWEKGPLMCRLRSRSAEAQ*
>*Cordyceps confragosa* RCEF 1005 TRI11
MLAGASNRNKLDTDHQYSQALAHVAYILLYNTFFHSLRKFPGPWLHSFTSIPHTRLTLSGSSHRKHLALHLKYGPVV
RIAPNILSFNHPDALKDLRGHRKAGEPEHSKDPVSIRFNPDNIVGSNREHHTRFRRALAHGFSSQAMLEQEPTFRLY
VDQLFARLQENCSNGAVAVDLAKWYTFTAFDMIGDLAFGESFGCLESSTYHPWVALAFESLKSLAFLIEIGRYPNM
DVLLKRLLPGGLLSKFAKNKELSIAKVQKRLDSGSNRPDFIGKITEGSRSKRTEMTFEELASNASVLIVAGSETSATLLS
AAIFFLTRSPKALALLTDEVRHAFGRKDDIGLINTQGLSYMQAVLEEALRMYPPVAGGGSARLVAKGGVQIADYFIP
ENALVENDMWALHHNPKYFTRPDDFVPERWLGDKAFKDDRLDSVKPFSIGPRNCIGMKYVSPKLCKCFAYHESST
RLITGSLAYSEMRMILARTIWEFDIRLAESSQNCISLIVPNRRRETARHQVPAPSSKATTDQGAGFASGQHRKEPSA
QPVATDFYELLSELRKVAAKGRPKKRVNRVAYNIAKLEVEVIDMRRERDELRDQLARKKAEAATLREMLKLPNSGRI
NAT*
>*Memnoniella echinata* JCM 22618 TRI22
MAGLYLQVVALVVGIYVLSIPAQAIYNLYLHPLRKIPGPRLWIAFPILGQIARVRGVLDSYMCELHRIYGEAVRYGPD
EVSIITEQAWRDIYDHRPNQLERNILSSTRRPDIFDANEIDHDRYRKAMSHAFSPKGLQEQEPIVKEYVGMLIERLN
QVAAKDGKTDMVQWYNFTLFDLIGDLAFGQSFGGLRDQVLHFTISFTFEAFKLLTYLEAGARYPLLLKLLELFTPKSII
EARDRKEEHAEATVKLRLENGSMHGRGDFMDSMLRNRGKPQGLTDRELVANASTLITAGSETTATILCGMTYWLL
RTPEMYEKVVEEVRNAYNSESEILMNTTTTRLPFMIACFQEAFRLYPPVPSCLQRVTPETGITKISGYDLPPNTKVGV
HALAAYTDPRNWHNPDQYLPERWLPEAKTNPSSPYYNDRRSALQPFSVGPRSCIGRNMAEQEIRFILARLLWNFD
FELCPESRGWNDQKTHYLWEKHPLMCKVKARVF*
>*Monosporascus cannonballus* CBS 586.93 TRI22
MTLLLLQAAVLAAAIYVLSIPVQAFYNLYIHPLSKIPGPNLWIAFPILGQIARVRGVLDAYMCELHRTYGEAVRYGPD
EVSIITEQAWRDIYDHRPNQLERNILSTTRRPDIFDANEIDHDRYRKAMSHAFSPKGLQEQEPIVKGYLDMLIERLN
QVAAKGEKTDMAQWYNFTLFDIIGDLAFGQSFGGLRDQVLHFSISFTFEAFKLLTYLEAGARYPLLLKLLELFTPTSIIE
ARDRKEEHAEATVKQRLENGSMHGRGDFMDAMLRNRGKPQGLTDRELVANASTLITAGSETTATILSGMTYWLL
RTPDIYERVVKEVRSAYNYDSEILMNTTATRLPFMIACFQEAFRLYPPVPSCLQRVTPETGITRISGYDIPPNTKVGVH
ALAAYTDPKNWHSPDQFLPERWLPEAKDQSSPYYNDRRSTMQPFSVGPRSCIGRNMAEQEMRLVLARLLWNFD
LELCPESNNWKDQKTHYLWEKHPLMCKVKSRKF*
>*Monosporascus ibericus* CBS 110550 TRI22
MTVLLLQAAILAAATYALSIPVQAFYNLYIHPLSKIPGPKLWIAFPILGQIARVRGVLDAYMCELHRTYGEAVRYGPDE
VSIITEQAWKDIYDHRPNQLERNILSTTRRPDIFDANEIDHDRYRKAMSHAFSPKGLQEQEPIVKGYLDMLIERFNQ
VAAKGEKTDMVQWYNFTLFDIIGDLAFGQSFGGLRDQVLHFSISFTFEAFKLLTYLEAGARYPLLLKLLELFTPKSIIEA
RDRKEEHAEATVKQRLENGSMHGRGDFMDAMLRNRGKPQGLTDRELVANASTLITAGSETTATILSGITYWLLRT
PDIYEKVVKEVRSAYKSDSEILMNTTTTRLPFMIACFQEAFRLYPPVPSCLQRVTPETGITQISGYDIPPNTKVGVHAL
AAYTDPKNWHSPDRFLPERWLPQAKNDPSSPYYNDRRSTLQPFSVGPRSCIGRNMAEQEMRLILARLLWNFDLEL
CPESNNWQDQKTHYLWEKHPLICKVKSRSF*
>*Paramyrothecium roridum* NRRL 2183 TRI22
MYALLVKAAVFAAAFYVLSIPAQAIYNLYFHPLRHIPGPKLWIAFPILGQISRVRGVLDSYMCELHRIYGEAVRYGPD
EVSIITEQAWRDIYDHKPNQLERNILSSTRRPDIFDANEVDHDRYRKAMSHAFSPRGLQEQEPIVKGYLDMLIDRLK
HVAEKGEKTDMVQWYNFTLFDIIGDLAFGQSFGGLRDQVLHFSISFTFEAFKLLTYLEAGARYPLLLKLLELFTPKSIIE
ARDRKEEHAEATVKQRLENGSMHGRGDFMDAMLRNRGKPQGLNDKELVANASTLITAGSETTATILCGVTYWLL
RSPDIYEKVVQEVRSAYNSESEIQMSTTITKLPFTVACIQEAFRLYPPVPSCLQRVTPETGITRISGYDIPPNTKVGVHA
LAAYTDPMNWHKPELFLPERWLPEAKKDESSPYYNDRRNALQPFSVGPRSCIGRDMAGQEMRLILARLLWNFDL
ELCPESNDWNDQKTHYLWEKHQLMCKVKNRVF*
>*Spicellum ovalisporum* DAOM 186447 TRI22

MAITLIQGAALVAAIYVLSIPAQAIYNLYFHPLSHIPGPKLWIAFPILGQICRIRGLLEARMCELHRVYGEAVRYGPDEV
SIITEQAWKDIYDHRPNQLERNILSTTRRPDIFDAEEDNHNRYRKAMSHAFSPKGLQEQEPIVKSYLDLLIERLNQVV
AKGEKADMVQWYNLTLFDIIGDLAFGQSFGGLRDQVLHFTISFTFEAFKLLTYLEAGARYPLLLKALELITPKHIIDAR
DRKEEHAEATVKKRLENGSMHGRGDFMDAMLKNHGKPQGLNEKELIANASTLITAGSETTATILSGTTYWLLRNP
EIYDKLATEVRTAYSSESEIQMITTASKLPFMSACFQEAFRLYPPVPSCLQRVTPEGGNTNVSGYELPPKTKVGVHAL
SAYTHPMNWHKPEQYLPERWLPEAKNSPTSPFYNDRRSSLQPFSVGPRSCIGRNMAEQEMRLILARLLWNFDFEL
CPESNDWKNQKTHYLWEKQPLWVKVKSRKS*
>*Spicellum roseum* DAOM 209012 TRI22
MAITLIQGAALVAAIYVLSIPAQAIYNLYFHPLSHIPGPKLWIAFPILGQICRIRGLLEARMCELHRVYGEAVRYGPDEV
SIITEQAWKDIYDHRPNQLERNILSTTRRPDIFDAEEDNHNRYRKAMSHAFSPKGLQEQEPIVKSYLDLLIERLNQVV
AKGEKADMVQWYNLTLFDIIGDLAFGQSFGGLRDQVLHFTISFTFEAFKLLTYLEAGARYPLLLKALELITPKHIIDAR
DRKEEHAEATVKKRLENGSMHGRGDFMDAMLKNHGKPQGLNEKELIANASTLITAGSETTATILSGTTYWLLRNP
EIYDKLATEVRTAYSSESEIQMITTASKLPFMSACFQEAFRLYPPVPSCLQRVTPEGGNTNVSGYELPPKTKVGVHAL
SAYTHPMNWHKPEQYLPERWLPEAKNSPTSPFYNDRRSSLQPFSVGPRSCIGRNMAEQEMRLILARLLWNFDFEL
CPESNDWKNQKTHYLWEKQPLWVKVKSRKS*
>*Stachybotrys chartarum* IBT 7711 TRI22
MTVLLQAAALAAAAYTLSIPIQSIYNLYFHPLSKIPGPKLWIAFPILGQIARVRGVLDSYMCAFHRVYGEAVRYGPDE
VSIITEQAWKDIYNHRPNQLERNILSTTRRPDIFDAVEVDHDRYRKAMSHAFSPKGLQEQEPIVKGYLDLLIERLNQV
AANEGKTDMVQWYNFMLFDTIGDLAFGQSFGGLRDQVLHFSISFTFEAFKLLTYMEAGARYPLLLKVLELFTPKSIIE
ARDRKEEHAEATVKQRLENGSMHGRGDFMDAMLRNRGKPGGLNDRELIANASTLITAGSETTATILSGMTYWLL
RNPDNYKKVVHEVRSAYSSDSEILMITTTTRLPFMIACFQEAFRLYPPVPSCLQRVTPETGMTQISGYDIPPNTKVGV
HALAAYTDPRNWHRPDEFLPERWLPEVEKNPASPFYKDRRATLQPFSVGPRSCIGRNMAEQEMRLILARLLWNFD
LALCPESKDWKEQKTHYLWEKHPLMCSVRRRVF*
>*Stachybotrys chlorohalonata* IBT 40285 TRI22
MTVLLQAAALAAAVYTLSIPIQSIYNLYFHPLSRIPGPKLWIAFPILGQIARVRGILDSYMCAFHRVYGEAVRYGPDEV
SIITEQAWKDIYNHRPNQLERNILSTTRRPDIFDAVEVDHDRYRKAMSHAFSPKGLQEQEPIVKGYLDLLIERLNQVA
ANEGKTDMVQWYNFMLFDTIGDLAFGQSFGGLRDQVLHFSISFTFEAFKLLTYMEAGARYPLLLKFLELFTPKSIIEA
RDRKEEHAEATVKQRLENGSMHGRGDFMDAMLRNRGKPGGLNDRELIANASTLITAGSETTATILSGMTYWLLR
NPDNYKKVVHEVRSAYSSDSEILMITTTTRLPFMIACFQEAFRLYPPVPSCLQRVTPETDMTQISGYDIPPNTKVGVH
ALAAYTDPRNWHRPDEFLPERWLSEAEKNPASPFYKDRRATLQPFSVGPRSCIGRNMAEQEMRLILARLLWNFDL
ALCPESKNWKEQKTHYLWEKHPLMCNVRRRVF*
>*Trichoderma arundinaceum* IBT 40837 TRI22
MANAISVGVAVQLVLTVLLASIPLRVIWNLFFHPLSYIPGPRLWIAFPIFRQIASIRGVFDAQMCEYHRKYGNAVRFS
PNEVSFITEQAWRDIYDHRPNQLERFILSTTRRPDIFDANEIDHARYRKAMLPAFSPKGLQEQEPIVRGYIDTFIERLR
EVSATGESTDMVKWYNFTTFDIIGDLAFGESFGGLRNREYHFTISFTFEAFKLLSYLEAGAAYPLLLKILMAFTPQSLIE
ARDKKEEHAETTVRKRLDNRALHGRGDFMDYLLRNRGEKQGLNDKELVANASTLITAGSETTATILSGITYWLLQTP
NVLQKVTEEVRSAFQSEADITFTSATSQLPYMLACFQEAFRHYPPVPTGMPRVTPSHGITKISGYDISPNTKVSVHQ
LAAYSHPDNFHRPREFVPERWLPDAKTNPSSPWYNDRRETVQPFNVGPRNCVGRNLAEQEIRVMLARVLWNFD
LELAPESKNWTDQKTHFLWEKGALMCKLHDRFASK*
>*Trichoderma brevicompactum* IBT 40841 TRI22
MANAISVSVAVQLVLTILIASIPIRVIWNLFFHPLSYIPGPRLWIAFPVFRQIASIRGVFDAQMCEYHRKYGNAVRFSP
NEVSFITEQAWRDIYDHRPNQLERFILSTTRRPDIFDANEIDHARYRKAMLPAFSPKGLQEQEPIVRGYIDTFIERLRE
VSATGESTDMVKWYNFTTFDIIGDLAFGESFGGLRNREYHFTISFTFEAFKLLSYLEAGAAYPMLLKILMAFTPQSLIE
ARDRKEEHAETTVRKRLDNRALHGRGDFMDYLLRNRGEKQGLNDKELVANASTLITAGSETAATILSGITYWLLQT
PNVLRKVAEEVRSAFQSETEITFTSATSQLPYMLACFQEAFRHYPPVPTGMPRVTPSHGITKISGYDIPPNTKVSVHQ
LAAYSHPDNFHRPQEFAPERWLPEAKTNPSSPWYNDRRETVQPFNVGPRNCVGRNLAEQEIRVMLARVLWNFD
LELAPESKNWTDQKTHFLWEKGALMCKLHDRFATK*
>*Trichoderma psychrophilum* S647 TRI22
MANPISTGVALQVLLSIFFISIPIRIIWNVSFHRLSYIPGPKLWIAFPILRHIASIRGQLDALMCEFHLKYGNAVRFGPDE
VSFITDQAWRDIYDHRPNQLERFILSTTRRPDIFDANEVDHSRFRKAMLPAFSPKGLQEQEPIVKGYIDMFIDRLKEV
AAAGKPTDMVQWYNYTTFDIIGDLAFGESFGGLRNQEYHFTISFTFEAFKLLTFLEAGAAYPLLLKMLMFFTPQSVIE

ARDKKEEHAETTVRKRLDNRALHGRGDFMDTLLRHRGEKQGLSDKELVANASTLITAGSETTATILSGITYWLLRNH
TTLQKVTQEVRSTFESEDDITFNAAASQLPYMAACFQEAFRLYPPVPSGLPRVTPSPGLTRISGYDIPPNTKVSVHQL
AAYSHSNNFHRPEQYVPERWLPEAKTDPSSPWYGDHREVLQPFNVGPRNCIGRNLAEQEIRVMLARVLWNFDLE
LCPESEQWTKQKTHYLWEKGALMCKLKNRATAK*
>*Trichothecium roseum* DAOM 197141 TRI22
MGNNLSVTTAVGLVAATAILQIPLRIIYNLFLHPMASIPGPKLWIAFPMLRQIANIGGTLDARMREYHLHYGSVVRF
GPDEVSFITEQAWRDIYDHRPNQLERFILPTTKRPDIFDADEHDHARHRKVMQPAFSPKGLQAQEPIVRGYVDLLV
KRMRELADKGEKADMVKWYNFTTFDIIGDLAFGEPFGGLRDAIYHFTISFTFEAFKLLSFLEAGASYPILLKILMAFTP
QSLIDARDKKEEHAETTFRKRLETEALHGRGDFMDTLLRNRNEKNGLNDKELIADASTLITAGSETSATVLSGVTYYL
LTNPDILQKVKDEVRTTFKQESDICFTEAAKKLPYMIACFQEGLRMYPPVPTGLQRVTPSSGITKIAGLDIPPNTKVSV
HQSAAYTHPDNFHRPTEFIPERWLPDAKTNPSSPFYKDHRDAVQPFSVGPRNCIGRNLAEHEMRVIMACVMWN
FDLELCPESKNWKDQKSHFLWEKGPLMCKIKNRVFA*
>*Trichoderma rodmanii* CBS 121553 TRI22
MAIVISTGVALQLLITIFLASIPLRVIWNLFLHPLRYIPGPRLWIAFPVFRQIASIRGVFDAQMCEYHRKYGSAVRFSPN
EVSFITEQAWRDIYDHRPNQLERFILSTTRRPDIFDANEIDHARYRKAMLPAFSPKGLQEQEPIVRGYIDTFIERLREV
SASGESTDMVKWYNFTTFDIIGDLAFGESFGGLRNREYHFTISFTFEAFKLLSYLEAGAAYPLLLKILMAFTPQSLIEAR
DKKEEHAETTVRKRLDNHALHGRGDFMDYLLRNRGEKQGLNDKELVANASTLITAGSETTATILSGITYWLLQTPD
VLQKVTREVRSAFQSEVDITFTSATAQLPYMLACFQEAFRHYPPVPTGMPRVTPSVGITRISGYDIPPNTKVSVHQL
AAYSHPDNFYRPREFIPERWLPEAKADRSSPWYNDRRETVQPFNVGPRNCVGRNLAEQEIRVMLARVLWNFDLE
LAPESKNWTDQRTHFLWEKGALMCKLSDRFATK*
>*Trichoderma protrudens* CBS 121320 TRI22
MANAISVGVAVQLVLTILLASIPLRVIWNLFFHPLSYLPGPRLWIAFPVFRQIASIRGVFDAQMCEYHRKYGNAVRFS
PNEVSFITEQAWRDIYDHRPNQLERFILSTTRRPDIFDANEIDHARYRKAMLPAFSPKGLQEQEPIVRGYIDTFIERLR
EVSATGESTDMVKWYNFTTFDIIGDLAFGESFGGLRNREYHFTISFTFEAFKLLSYLEAGAAYPLLLKILMAFTPQSLIE
ARDRKEEHAETTVRKRLDNRALHGRGDFMDYLLRNRGEKQGLNDKELVANASTLITAGSETTATILSGITYWLLQTP
DVLRKVTEEVRSAFQSEADITFTSATSQLPYMLACFQEAFRHYPPVPTGMPRVTPSNGITKISGYDIPPNTKVSVHQL
AAYSHPDNFHRPRDFIPERWLPDAKTNPSSPWYNDRRETVQPFNVGPRNCVGRNLAEQEIRVMLARVLWNFDLE
LAPESKNWTNQKTHFLWEKGALMCKLHDRFATK*
>*Trichoderma turrialbense* CBS 112445 TRI22
MANAISVSVAVQLVLTILIASIPIRVIWNLFFHPLSYIPGPRLWIAFPVFRQIASIRGVFDAQMCEYHRKYGNAVRFSP
NEVSFITEQAWRDIYDHRPNQLERFILSTTRRPDIFDANEIDHARYRKAMLPAFSPKGLQEQEPIVKGYIDTFIERLRE
VSATGESTDMVKWYNFTTFDIIGDLAFGESFGGLRNREYHFTISFTFEAFKLLSYLEAGAAYPLLLKILMAFTPQSLIEA
RDRKEEHAETTVRKRLDNRTLHGRGDFMDYLLRNRGEKQGLNDKELVANASTLITAGSETTATILSGITYWLLQTPN
VLRKVTEEVRSAFQSETEITFTSATSQLPYMLACFQEAFRHYPPVPTGMPRVTPSHGLTKISGYDIPPNTKVSVHQLA
AYSHPDNFHRPQEFVPERWLPEAKTNPSSPWYNDRRETVQPFNVGPRNCVGRNLAEQEIRVMLARVLWNFDLEL
APESKNWTDQKTHFLWEKGALMCKLHDRFATK*
>*Trichoderma taxi* ZJUF0986 TRI22
MANTISASLALQLVVTIFIASIPLRVIWNLFFHPLSYIPGPKLWIAFPILRQIASIQGAFDAKMCDYHRKYGNAVRFSP
NEVSFITEQAWRDIYDHRPNQLERFILSTTRRPDIFDANEMDHARYRKAMLPAFSPKGLQEQEPIVKGYIDTFIERLK
EVSNSGKPTDMVKWYNFTTFDIIGDLAFGESFGGLRNREYHFTISFTFEAFKLLSYLEAGAAYPLLLKVLMAFTPQSLI
EARDKKEEHAETTVRKRLDNRALHGRGDFMDYLLRNRGEKQGLNDKELVANASTLITAGSETTATILSGITYWLLQT
PVVLQKVTEEVRSTFQSEAEITFTSATARLPYMLACFQEAFRHYPPVPTGMPRVTPSTGMTRISGYDIPPNTKVSVH
QLAAYSHPDNFHRPQEFIPERWLHEAKTDPTSPWYNDRRETVQPFNVGPRNCVGRNLAEQEIRVMLARVLWNF
DLELAPESKNWTDQKTHFLWDKGALMCKLTDRFDT*
>*Trichoderma albolutescens* CBS 119286 TRI22
MANAISAGVALQLVITIFLASIPLRAIWNLFFHPLSYIPGPKLWIAFPVFRQISSIRGVFDAQMCEYHRKYGNAVRFSP
NEVSFITEQAWRDIYDHRPNQLERFILSTTRRPDIFDANEVDHSRYRKAMLPAFSPKGLQEQEPIVKGYIDTFIERLRE
VSATSKPTDMVKWYNFTTFDIIGDLAFGESFGGLRNREYHFTISFTFEAFKLLSYLEAGASYPLLLKILMAFTPQSLIEA
RDKKEEHAETTVKKRLENRALHGRGDFMDYLLRNRGEKQGLNDKELVANASTLITAGSETTATILSGITYWLLQTPK
VLEKVTQEVRSTFQTEEDITFTSATAQLPYMLACFQEAFRHYPPVPTGMPRVTPSPGITRISGYDIPPNTKVSVHQLA

AYSHPDNFHRAKEFVPERWLPDAKTDPSSPWYNDKRETVQPFNVGPRNCVGRNLAEQEIRVMLARVLWNFDLEL
APESKNWTNQRTHFLWDKGALMCKLTDRFAK*
>*Trichoderma rubi* CBS 127380 TRI22
MSNTIPASLALQLVVSIFIASIPLRVIWNLFFHPLSYVPGPKLWIAFPILRQIASIQGVFDAQMCEYHQKYGNAVRFSP
NEVSFITEQAWRDIYDHRPNQLERFILSTTRRPDIFDANETDHSRYRKAMLPAFSPKGLQEQEPIVRGYIDTFIERLRE
VATSGKPTDMVKWYNFTTFDIIGDLAFGESFGGLRNREYHFTISFTFEAFKLLSYLEAGAAYPLLLKILMAFTPQSLIEA
RNKKEEHAETTVRKRLDDRALNGRGDFMDYLLRNRGEKQGLNDEELVANASTLITAGSETTATILSGITYWLLQTPA
VLQKVTEEVRSAFQSEDDITFTSATARLPYMLACFQEGFRHYPPVPTGMPRVTPSTGMTRISGYDIPPNTKVSVHQ
LAAYSHPDNFHQPQEFMPERWLHEAKTDPLSPWYNDRRETVQPFNVGPRNCVGRNLAEQEIRVMLARVLWNF
DLELSPESKNWANQKTHFLWEKGALICKLTNRCARN*
>*Microcyclospora tardicrescens* HJS 1936 TRI22
MPTSIVLVALFVGSYLVYAVGRAVYDLYLHPLRSVPGPKLWIVFPSLRQYANIRGDLDAEMRKLHLKYGEAVRFGPN
EVSFITEQAWKDIYDHRPNQLERSILSDTRRPDIFDANEIDHDRYRKAMAPAFSPRGLISQEPIIKGYIDQFVDRLKEV
AAAGAAVDILKWYNLTTFDIIGDLAFGEPFGGLKNQEYHFTISFTFEAFKLLTYLEAGAAYPLLLKLLMMFTPKSLLEA
RDRKEEHAAETVQKRLKNGKLHGRGDFMDSMLRHRGEKQGLSDIELVANASTLITAGSETTATLLSGMTYWLLQN
PAAMAKVTSEVREAFANEAEIGFNSTVTKLPYMLACIEEGLRLYPPVPSGLQRVTPAVGMTRISGLEIPPNTKVSVH
QSAAFTHPDNWHNPDQFIPERWLAKSDPTSPYYNDRREILQPFSVGPRSCPGKNLAYHEIRTIFARILYRFDLSLTPE
SADWNVQKSYFLWEKTPLMINLKDRL*
>*Fusarium camptoceras* NRRL 13381 TRI11
MFHHFIWPFAGVAAGICLTYLVVVVVYNLFFHPLRHFPGPWLNRTTQIPHTLLMLCGLPHKKHLELHLKYGPVVRI
GPNMLSFNHPDAMKDVRGHRKAGEPEHGKDPISVQFAGDNIVGSDRENHTRFRRALAYGFSAQAMLEQEPTFK
SYVNQLFQQLHEQSSGGKKTVDIAKWFTFTTFDMIGDLAFGESFSCLDNSTYHPWVALAFESLKSVAFLAEIGRYPS
LAPLAGFMLPRGLLTKFAENKELASMKVKKRLDTKTDRPDFVGKITQGLKSKGSSMKFDELASNASVLIVAGSETTA
TLLSAAVYFLCANRRTLDLLTQEVRTTYSQAKDIDLISTQNLRYMQAVLDEALRMYPPVAGGGSPRKIAEGGSHVA
GYYVPENTLVENDMWALHYDPKYFTQPHNFIPERWLGDSRFKDDRLDAVKPFSIGPRNCIGMNLAYAEMRMML
ARTVWEFDLRLAEDSRNWYKESRVYLAWHKPALNVYLTPR*
>*Fusarium flagelliforme* FIESC 12 MPI-CAGE-AA-0113 TRI11
MLHYSVWPFVAFTAGTCLTYLVAVLVYNLFFHPLRHFPGPWLNRTTQIPHTLLMLCGLPHKKHLELHLKYGPVIRIG
PNMLSFNHPDAMKDVRGHRKAGEPEHGKDPISVQFAGDNIVGSDRENHTRFRRALAYGFSAQAMLEQEPTFKSY
VNQLFQQLHEQSLGGKRTVDIAKWFTFTTFDMIGDLAFGESFSCLDNSTYHPWVALAFESLKSVAFLAEIGRYPSLA
PLAGLMLPRGLLTKFAENKELASMKVRKRLDTKTDRPDFVGKITQGLKSKGSSMKFDELASNASVLIVAGSETTATLL
SAAVYFLCANPRTLDLLTQEVRTTYAQAKDIDLISTQNLRYMQAVLDEALRMYPPVAGGGSPRKIAKGGSLVAGYY
VPENTLVENDMWALHYDPKYFTRPHEFIPERWLGDPRFKDDRLDAVKPFSIGPRNCIGMNLAYAEMRMMLART
VWEFDLLLAEDSRNWYEESRVYLAWHKPALNVYLTPR*
>*Fusarium graminearum* PH-1 TRI11
MFQYSLWPLLALSGGTAVAYFLVMLVYNLFFHPLRNYPGPWLNTMTQIPHTLLMLCGLPHKRHLALHMKYGPVV
RIGPNMLSFNHPDAMKDVRGHRKSGEAEHGKDPIIVLSNGDNIVGSDRENHTRFRRALAYGFSAQAMLEQEPTFK
AYVNQLFQRLHEQSSSGIKPVDISKWYTFTTFDMIGDLAFGESFGCLDNSTYHPWVALAFESLKSLAFMAEMGRYP
RIAPYIGFLLPRGLLTKFAENKELASMKVRKRLDTETDRPDFVGKITQGLKAKGSRMEFNELASNASVLIVAGSETTA
TLLSAAVYFLCSNPRTLELLTQEVRSTYTQADAIDLVSTQGLRYMQAVLDEALRMYPPVAGGGSPRKIAKGGSFVAG
YFVPEDTLVENDMWAMHYDPKYFTRPNDFIPERWLGDARFSSDRLDAVKPFSIGPRNCIGMNLAYAEMRMMLA
RTVWEFDIRLAESSRDWYQDSRVYLAWNKPPLNVYLDPR*
>*Fusarium longipes NRRL* 20695 TRI11
MMEHTIWRLLAFSAGTWLVYLATVLMYNLFFHPLRKFPGPWLNSITQIPHTLLMLCGLPHKKHLALHMKYGPVIRI
GPNMLSFNHPDAMKDVRGHRKSGEAEHGKDPISVQSNGDNIVGSDRENHTRFRRALAYGFSAQAMLEQEPTFK
AYVNQLFQRLHEQSCGGTSTVDISKWYTFTTFDMIGDLAFGESFSCLDNSTYHPWVSLAFESLKSLAFLAEIGRYPKI
APLLGLMVPRGLLTKFAENKELASMKVRKRLDTETNRPDFVGKITQGLKSKGSTMEFNELASNASVLIVAGSETTAT
LLSAAVYFLCDNPRTLELLAREVRSTYTQASDIDLVSTQGLRYMQAVLDEALRMYPPVAGGGSPRKIAKGGSFVAGY
FVPEGTLVENDMWALHYDPKYFTQPHDFIPERWLGDVRFENDRLDAVKPFSIGPRNCIGMNLAYAEMRMMLAR
TVWEFDISLADSSRDWYEQSRVYLAWNKPPLNVYLNPR*
>*Fusarium poae* FPOA_05388 TRI11

MFQFSLWPFLALSGGTWLAYVVMLLVYNLFFHPLRKFPGPWLNSITQLPHTLLMLCGLPHKRHLALHMKYGPVVR
IGPNMLSFNHPDAMKDVRGHRKSGEAEHGKDPISVQSNGDNIVGSDRENHTRFRRALAYGFSAQAMLEQEPTFK
AYVNQLFQRLHEQSFNGTKTVDISKWYTFTTFDMIGDLAFGESFSCLDNSTYHPWVSLAFESLKSLSFLAEIGRYPTI
APYAGLLVPRGLLTKFAENKELASMKVRKRLDTETDRPDFVGKITQGLKSKGTSMEFNELASNASVLIVAGSETTATL
LSAAVYFLCANPRTLDLLTEEVRSTYTRADDIDLVSTQGLRYMQAVLDEALRMYPPVAGGGSPRKIAKGGSFVAGYF
VPEDTLVENDMWATHYDPKYFNQPHDFIPERWLGDERFKNDRLDAVKPFSIGPRNCIGMNLAYAEMRMMLART
VWEFDIHLSKSSQNWYKESRVYLAWNKPPLEVYLVPR*
>*Fusarium sporotrichioides* NRRL 3299 TRI11
MFQYSLWPLLALSGGTGLAYLVVVVVYNLFFHPLRNFPGPWLNSITQVPHTLLMLCGLPHKKHLALHMKYGPVVR
IGPNMLSFNHPDAMKDVRGHRKSGEPEHGKDPISVQSNGDNIVGSDRENHTRFRRALAYGFSAQAMLEQEPTFK
AYVNQLFQRLHEQSSGGTKPVDISKWYTFTTFDMIGDLAFGESFSCLDNSTYHPWVSLAFESLKSLAFLAEIGRYPRI
APYLGLLVPRGLLTKFAENKELASMKVRKRLDTETDRPDFVGKITQGLKSKGTSMEFNELASNASVLIVAGSETTATL
LSAAVYFLCAHPRTLDLLTKEVRSTYTQAHDIDLVSTQGLRYMQAVLDEALRMYPPVAGGGSPRKIAKGGSFVAGH
FVPENTLVENDMWAMHYDPKYFTQPHDFIPERWLGDVRFANDRLDAVKPFSIGPRNCIGMNLAYAEMRMMLA
RTVWEFDIRLSEGSRNWYEESRVYLAWNKPPLNVYLIPR*
>*Calonectria aciculata* CMW 47645 TRI11
MFQYSIWPWLAFLVGTSLAYLVSVLVYNLFFHPLRKFPGPWLNSATQIPHTLLMLCGIPHKKHYALHMKYGPVVRI
GPNMLSFNHPDAMKDVRGHRKAGEPEHGKDPISVQFAVDNIVGSDRENHTRFRRALAYGFSAQAILEQEPTFKAY
VDQLFQRLHEQSSGGKKTVDIAKWYTFTTFDMIGDLAFGESFSCLDNSTYHPWVALAFESLKSLAFLAEMGRYPRL
APLASRMVPQGLLTKFAENKNLASMKVRKRLDTETDRPDFVGKITQGLKSKGGCLEFHELVSNVSVLIVAGSETTAT
LLSAAVYFLCTNPRTLALLTQEVRSTYAQASDIALISTQGLRYMQAVLDETLRMYPPVAGGGSPRKIAKGGSVVAGY
YIPENTLVENDMWALHYDPKYFTKPHDFTPERWLGDARFKNDRLDAVKPFSIGPRNCIGMNLAYAEMRMMLAR
TVWEFDISLAESSRNWYQQSRVYLAWHKPALDVYLTPR*
>*Calonectria fujianensis* CMW 27257 TRI11
MFQYSIWPWLAFLVGTSLAYLVSVLVYNLFFHPLRKFPGPWLNSATQIPHTLLMLCGIPHKKHYALHMKYGPVVRI
GPNMLSFNHPDAMKDVRGHRKAGEPEHGKDPISVQFAVDNIVGSDRENHTRFRRALAYGFSAQAILEQEPTFKAY
VDQLFQRLHEQSSGGKKTVDIAKWYTFTTFDMIGDLAFGESFSCLDNSTYHPWVALAFESLKSLAFLAEMGRYPRL
APLASRMVPQGLLTKFAENKNLASMKVRKRLDTETDRPDFVGKITQGLKSKGGCLEFHELVSNVSVLIVAGSETTAT
LLSAAVYFLCTNPRTLALLTQEVRSTYAQASDIALISTQGLRYMQAVLDETLRMYPPVAGGGSPRKIAKGGSVVAGY
YIPENTLVENDMWALHYDPKYFTKPHDFTPERWLGDVRFKNDRLDAVKPFSIGPRNCIGMNLAYAEMRMMLAR
TVWEFDISLAESSRNWYQQSRVYLAWHKPALDVYLTPR*
>*Fusarium sporotrichioides* NRRL 3299 TRI13
MFLSLCLMVLALYLLYKWALPKPISSIPYNPPALQSLFGDIPAMIQGTKANNQTHMDWIIQQMKNHESPIIQLFLSP
LQRPTVILADFRETQDIMLRRKDFDRSTNIRGLLEDVIPDHHIYEQTNSVFRTHRKLVQDVMLPSFIQKVAGPAFHS
NIMRLVRVWDLKAQIADGSPFLATQDIQGAVLDAVYSFAFGSYYKSSTTLPKIEKLEKWNGNAENSSRNAPQSDKP
FDFPDVAFDDLINATIDLAKAPQGLQGSPIAKLQAKVTMNMPHFRRVRKIRDDFLRGSLKSAVSKLPSEGGKSDSQ
AVTSAVEQMVLRETALAQTENRSPNYFSTMMQGELFGLILGGFDTTSTTTLWGLKFLTDNASVQKRLRQALQSSF
TKAKAENRSPTFQELAVARIPYLEAVIEEILRCAGATPALQRLAKVDTQILGYHIPKGTDVLFLTHGPSVWTPGFEIDE
SRRSQTCQAAGEKKDQCWESHDISKFKPERWLGQKLPSNNREKDTDATETAEEFDGLAGPTLAFGLGTRGCFGRR
LGYQQLKTSITILIWNFELLPCPQELSSYRTIEGLTSMPEHSYISLAKVDLTTT*
>*Fusarium longipes* NRRL 20695 TRI13
MFISLGLAVLAFYLLYKWALPKPISNIPYNHPALHSLFGDIPAMIRETKARDQTHMDWIIQQMKNLESPIIQLFLSPL
HRPTVILADFRETQDIMLRRKDFDRSTNIRGLLEDVIPDHHIYQQTNSVFRAHRKLVQDVMLPSFIQKVAGPAFHA
NIMRLVRVWELKSIIAKESPFLITQDIQGAVLDAVYSFAFGDYYVSSTTLPKIEILREWANAAADKSRGPFNRDKPFEF
PDVAFDALINATIDLAKAPQGLQGSPIAKLLAKVKMNMPHFRRVRRIRDNFLHGSIQSAVVKLSNRDEKSDCVSVTS
ALDQMILRETTLARAESRSPNYFSTMMQGELFGLILGGFDTTSTTTLWGLKFLTDNPLVQKRLRKALQSSLTTAKAE
GRSPTFQELAVARIPYLEAVVEEILRCAGATPALQRLTTVDTQILGHHIPKGTDVLFLTHGPSVWSPGFDIDENSRSQ
SCQIAREKKDQRWGSHDIAKFKPERWLLPKVSPTMDDKESDATVIAKEFDGSAGPTLAFGLGTRGCFGRRLGYQQ
LKTSISILIWNFELLACPPELSSYRTIEGLTSMPEHSYIRLAKVGSRTAV*
>*Calonectria aciculata* CMW 47645 TRI13

MFISVAVAVVALYLLYKWAHPRPISNIPYNTGALHSLFGDIPAMIQDIKANDQTHMDWIIQQMENLRSPIIQLFLSP
LQRPTVILADFRETQDIMLRRKEFDRSTNIRDLLGDVIPDHHIYQQTNLVFRAHRKLVQDVMLPSFIREVAGPAFHD
NIMRLTRVWEMKTTIANGSPFAATQDIQGAVLDAVYSFAFGENYKSSTTLPNIKILECLKSNSQQVAGGGEPLDFPE
VTFDDLINATMDLAKAPQGIQGSPIAKVTAKIKMNMPHFRKVRKIRDDFLRGSIESAVSKLLNKSERSDSSPVKSALD
QMVLRERILAQDENRSPNYYSSMMQGELFGLILGGFDTTSTTTLWGLKFLTDNSSVQKRLRNAMQSSFTLARAEN
RSPSFQELASARIPYLEAVIEEILRCAGATPALQRVSIVDTQILGYHIPKGTDVLFLTHGPSVWSPRFVIDESTRSQSCQ
VAGDKKDQRWDDFDIASFKPERWLVASSSSTEDAAETTQSFDATAGPSLAFGLGTRGCFGRRLGYQQLKTSIAILI
WNFELLPCPSKLSSYRTIEGLTSMPEHSYIRLAKVDLTTTE*
>*Calonectria fujianensis* CMW 27257 TRI13
MFISVAVAVVALYLLYKWAHPRPISNIPYNTGALHSLFGDIPAMIQDIKANDQTHMDWIIQQMENLKSPIIQLFLSP
LQRPTVILADFRETQDIMLRRKEFDRSTNIRDLLGDVIPDHHIYQQTNLVFRAHRKLVQDVMLPSFIREVAGPAFHD
NIMRLTRVWEMKTTIANGSPFAATQDIQGAVLDAVYSFAFGENYKSSTTLPNIKILECLKSNSQQVAGGGEPLDFPE
VTFDDLINATMDLAKAPQGIQGSPIAKVTAKIKMNMPHFRKVRKIRDDFLRGSIESAVSKLLNKSERSGSSPVKSALD
QMVLRERILAQDENRSPNYYSSMMQGELFGLILGGFDTTSTTTLWGLKFLTDNSSVQKRLRNAMQSSFTLAKAEN
RSPSFQELASARIPYLEAVIEEILRCAGATPALQRVSIVDTQILGYHIPKGTDVLFLTHGPSVWSPRFVIDESTRSQSCQ
VAGDKKDQRWDDFDIASFKPERWLVASSSSTEDAAETTQSFDATAGPSLAFGLGTRGCFGRRLGYQQLKTSIAILI
WNFELLPCPSKLSSYRTIEGLTSMPEHSYIRLAKVDLTTTE*
>*Nannizzia gypsea* CBS 118893
LLFTFVKTVYNLYFHPLRSYPGPWLARASRWYYSYYLKIGRLPHKTKEWHDKYGRCVRIAPDELSYNTAEAWEDICG
HRTESRMDSFEKDLTFFPPSPNGVDSIVIHRRFRRLLSHPMSDKALSAQQEIITGYVDQLIDELRERSVGHKGDKKGV
VDMVRWFNFTSFDILGDLAFGEPFGCLRSGVMHPWIELIFTAIKSVMDMQIIRRVPGAFPLMMAIAGMFQQSQH
LQDQFMFCQKKARERLSRETTRPDFMTYILRATEEKGMTQDEIEANAQILIMAGSETTASALSGTLFYLLKSPEVMQ
KLRKEMECNFQQESDITMRSTQGLEYLNAVIQEAMRVYPPVPCTFPRTTPPAGAMVCGQFVPGGYIVGINQMAA
MTSAKNFTDPTKFVPERWLGDERYISDCKKAYQPFSYGPRNCLGKNLAYAEMRLVLTRLLWNFDLELLEESKDWH
DRQKVWMMWDKGDLNVKIRPL
>*Aspergillus clavatus* NRRL 1
LGYILASAIRNIYFHPLRDIPGPKSWIAFPLLQHLSAIRGRLDLDMHHWHIKYGAAVRFDPTSVSFITADAWRDIYGH
GHKQLPKVLNSGSNTQDIISANDADHTRHRKALAHAFSAKGLQAQEPIVTGYVDQLIARLRDVAASGLPADMVK
WYTLTTFDLIGDLAFGEPFGGLESSEYHRWVAAVFGFIRVIPFLKGMDEYSVLFRVILSFLPRSFLQMRTDQVEHTKR
TVQKRLRTRRHDRSDFMDSMLRHRGDKDGLSEEELVANANILIIAGSETTATLLSGVTYWLLQTPAAMERVTREVR
TTFASEAEITFNNVTAQLPYMLACISEAFRLYPPVPGGLQRWTETPTRISGYEIPGRTKVSVHQAGAYWSSRNFHQP
DSFIPDRWLPEAKDDPASSFFSDQRDVLQPFSVGPRNCIGKNLAYTEMRVILARVLYNFDLQLCEESRDWKDQKTF
VLWEKKPLMCRLTAR
>*Aspergillus oryzae* RIB40
LYSLLTAIWYIYFHPLRRIPGPKSWIIFPIMRHASAIRGRFDIDMRQHHAVYGPVVRFGRDEVSFITAEAWKDIYGHG
HQQLPKVLSSASNMLDIISSNDTDHSRYRKALSHAFSAKGLQAQEPLLNSYVDKLIERLKGIAESNLPADMVKWYNL
TTFDIIGDLAFGEPFGGLDNSEYHHWVSTIFESIKAIPFLKLKDAYPLAFKAILGLIPKGIMEARKRQLEHSRITVQKRLQ
TSSSYNRGDFMDSMLRNRGEKDSLNDSELEANSNILIIAGSETTATLLSGATYWILRNPEALAKLTDEVRSVMKSEPE
ITAQKASAELPYMLACFDEAFRLYPPVPTGLQRRTLVPTRISGCDIPAGTKVSVHQSAAYWSSTNFHAPDRFIPERW
LPEAKSDPSSPFYSDNRGVVQPFSTGPRNCIGRNLAYAEMRVILARVLWNFDLELCEESTQWSDQKAYTLWEKPPL
MCRLKLR
>*Aspergillus terreus* ATEG_08832
VLYIFLLAIWNLYFHPLRHIPGPRSWIAFPIMRHISASRGRLDSDMRRFHAQYGGAVRLAPAEVSFITPDAWKTIYG
HGHTQLPKVQTSESKGLDIISSEGPNHTRHRKALAHAFSARGLQAQEPLVRGYVDKLIERLKVDMVKWYNLTTFDLI
GDLAFGESFGGLDNSRVHSWVSTIFRSVKVLPFVRITDTYPILIPLLMALLPKSLRTARRDQTNYSKETVHKRLTAAH
GRGDFMDSMLRGEKDGLSDRELEENASILIIAGSETTATLLSGVTYWLLRSPEALAKVTDEVRSTFQTEGEITLQDVG
ALPYMLACLDEAFRMYPPVPCAERRVLTPIVIAGYNIPPGTVVSVHQSAAYCSPANFHRPQDYIPERWLPNPSYFSD
QRDVLQPFSVGPRNCIGKNLAYAEMRLILARVLWNFDLELCEESLHWKDQKSYLLWDKPPLMCKLKQR
>*Aspergillus niger* An11
IVYTVCAAIWNIYLHPLHRIPGPKLWLAFPILRYISFMRGVLDRDMRTYHNRYGGAVRFSPNEVSFITTQAWKDIYG
HGHRQLPKVPGSMNNPMDIISANDSDHTRFRKAISHAFSAKGLQAQEPFIMEYVDKLINKLHSMAKSQVAVDMA

KWYNLTTFDLIGDLAFGESFGGLDSTEYHYWVSTIFEFIKAIAFARFKDNYPMMFQVLERFLPKHLLEAKRRQDEYS
WNTVQKRLHHQRDRGQADFMQSMLRHRGEKDGLTDEELAANASILVIAGSETTATLLSGLTYWLLQHPKEMEKV
KFEVRSVMKTEEDIDVNNATAKLPYMLACIDEAFRMYPPVSGGLQRYVPDTPTEISGYLLPPKTNVSVHQSAAYRS
PLNFYRPEEFIPERWLPQSKNDPSSPFYHDNRDVLQPFSVGPRNCIGRNLAYAEMRVILARVLWNFDIELCEESRD
WHDQKSYNLWAKPPLMCRLKAR
>*Neurospora crassa* OR74A
VSSILLSAIYNLTLHPLARHPGPFFHRASILPYLYRQITGTLPCSILDFHARYGPVVRISPNALSFADPQAWKDIYGHRP
HGEEEFAKLNLFYRIKGSPPSLLSETKEAHGTLRKLMAHGFSDRSMRAQEGIIGGYVSALIRGLRGNCRSSSGKREDV
VKQNEGVAEEEEEEKETTAVINMKNTNTKQTENMNQKEEEQDERVPLDMVSWYNWTTFDIIGDLAFGEPFGCLE
KAEYDPWVDAVGKSVRFGCVMFALRLLGLEDWVCPLVRKLSGNARRFHRKRTMDKLQRRVKLTKERPDFLEGLL
QKREEWGIDMDALAANASLLIVAGSETTATLLAGATYMLLRDPEAMKKLTEEVRSTFKSEEEITLSSVGNLEYMLAC
LNEAMRLYPPVPIGMPRVVPKGGAKVAGTFVPEGTVVAVWHWATSHNEQHFVEPFEFHPERWMQDPRFAND
RLDAVQPFSVGPRNCIGRSLAIAEMRLILTKVVYNFDMKLANPEKDWLDQHIYTLWDKHPLPVYLTPI
>*Neurospora tetraspora* CBS 560.94
VSSILLSAIYNLYLHPLASFPGPLLHRASLFPYLYRQVTGKLPYSVLDFHARYGPVVRISPNSLSFADPQAWKDIYGHRS
PGQEEFAKPNLFYRTKGIPPSILSETRENHGVLRKLMAHGFSERSMRAQEGIIGGYVNALISGLRGHCFGKEEKKDD
GVSSEKETEQKKQQPVPLDMVSWYNWTTFDVIGDLAFGEPFGCLEKAEYDPWVDAIGKSVRFGSILLAIRVLGLED
VVHPIIEKLSNKARRFHRKRTMDKLQRRVNLTKERPDFLEGLLQKREEWGMDMERLAANASLLIIAGSETTATLLSG
ATYLLLKHPEAMKKLIEEVRSTFKSEEEITLSSVGSLEYMLACINEAMRLYPPVPIGMPRVVPKGGAKVAGTFVPEGT
VVAVWQWATNHNEQHFLEAFEFHPERWMHDPRFANDRLDAVQPFNVGPRNCLGRNLAIAEMRLILAKVVYNF
DMQLANPGKDWLDQQIFTFWDKPALPVCLTPV
>*Sordaria macrospora* k-hell
LYVFGYAIYNLYFHPLAKYPGPLLMRATRLGYCQRLLKGTLSFDLLELHKTYGDIVRVAPNELAFANSQAWKDIMGH
RTGPGGDFEKWEKFYRPVDGVPTDAGREEHGMLRRTMAHGFSDRSMRDQQPLIKGYIDLLMQRLRVDIAAWYN
FTTFDVIGDLAFGEGFGCLEKSEYHPWVKNLFKMASAGTIFQVLSHYPPLIKLMLALAPKSLMEEHEKHTENAKDKL
RRRMEMGHDRADLVEGLLKKDEWGLTLDKLQANSAILIIGGSETTATLLAGATFLLATSPVALKKLTDEIRSAFNSED
EIDYASVSVLPYLLACLDEALRMYPPVPTGLPRVVPKGGASITGHFIPEDSIVAIHQWAMYHNDKHFKDPFTFHPER
WLNDPAFENDHKEAFQPFHIGARNCLGKNLAYLEMRIILARLLWNFDLKIAEDSVDWMKKQKIFNFWDKDALNV
YLTPR
>*Glomerella graminicola* M1_001
LAYVFGTIVYNVYFHPLCNYPGPKLWAATRIPLARSALSGQLHRKILQLHQEYGPVVRVAPDELVFNHPDSFKDIHG
HVKNNTGDHGRDPVFIRGMEHGIIGANREDHTRFRRALSHGFAAQTMLEQQPIIIAYIDLMIQRLHLDMVSWYN
WTTFDVIGDLAFGESFHCLEDADYHPWVQLIFDSVKAGSVAITMRRFPWAAKILMKFVPAELIRKRAQHFAMTEQ
KVKKRLAAKTERPDFMDSMLRRGSEALTFDELKVNSSTLITAGSETTATALSAMTYYLLTNPNALKKASDEIRNTFTS
EADIDMISSQKLVYMHAVINEALRMYPPAPSGIPRRVKSDGGVFLGQYVPPDTIVHVWHWAMYHSPANFALPDS
FIPERWLDDPRFAGDKKEALQPFTVGPRSCIGRNLAYGEMRLILARVLFNFDLRLADESRGWDEQSQVYTLWEKGP
LKVFLTPR
>*Trichoderma atroviride* IMI_206040
LAYAFCITVYNLFFHPLRNYPGPWLWAASDIPYSLVSIGGNAHKKMLQIHIKYGPVVRVGPNTVFYSHPDASKELRG
HRKGKGVEHLKDPHLHSGNQSNAIGASRENHIRYRRNLSYGFSHQAMLDQEPIINRYINTLLTELKARCANQEKVD
VVRWYNYTTFDIIGDLAFGEPFYCLEKSDYHPWVALIFSGVKNMSFISVCSKFGKLGMLLALLFVPKDLPAKGREHR
RLSIEKTRRRLDSGSSRPDFMTALTTTRGSAEELSFEELVSNASLLIAAGSETTATALSAATYYLGIYPETFKKLAAEIRS
AFRSEEDITLTSAQHLNYLQGVIDEAMRLFPAAPGTQPRIISPGGDVIVGRYVPAGTVVGIWQWVNHHNPAHFRD
AESFIPERWLGDARFENDKRDAFMPFSVGPRNCIGHNLAYAEMRLILAKVVWNFDIELSEESIGWDTRSKVYMLW
EKGPINVQLTKR
>*Trichoderma reesei* QM6a
MCVTIYNLFFHPLSRYPGPWLWAVSDIPYSLVSISGDAHKRMLQIHMRYGPVVRVGPNTVFYSHPDATKEIRGHRK
GNKAEHLKDPHLHSGNQSNVIGANHENHVRYRRSLAHGFSHQAMLDQEPIINKYIDKLLKELKNQSTKQEKIDIVR
WYNYTTFDIIGDLAFGEPFYCLEKSDYHPWVALIFSGIKNLSFMSVCSKHGQLGKIVAMFLVPKDLPKKGTQHRRLSI
EKVRRRLDSGSSRPDFMTAMMTPRGSSEELTFTELASNASLLIAAGSETTATALSAATYYLGLNPETFAKLAAEVRSV
FCSEKEITLTNVQHLSYLQAVIDEAMRLFPSAPGTQPRIISPGGDTIVGRYVPEGTVVGVWQWVNHHNPAHFRDP

ESFLPERWLGDARFESDKRDAFMPFSIGPRNCIGRNLAYSEMRLILARMVWSFDIRLAEESVGWDMRSKVYMLW
EKGPIYVYLTRRE
>*Diaporthe citri* NFHF-8-4
MSRAPLPALSLEQGVTNVNSSNQPYTLAFAIVGLLLYAFYRWALPKPISDIPYNESGRKNVLGDIPSLLEGTQRTGEF
NLWLLEQESKLRAPLFQVFIRPLGRPMLVMCDFRESQDILMRRKDFDRSSLLVDTLGGIGPDHHIVLKTDAEWKRH
RRLVQDLMSPAFLNEVAGPTVYKGVLKLIDLWSDKARLADGRPFSAETDIYHAALDAVMAFTFGGSFPSSATGPM
VDAVKALGAEDIKKLGGNGVDDPIVFPEGKSDEKIRATLDVPGAIEHVQGSPMPKLKWWLVKKRPDFRKAFSIKNA
YVQEEISKSLRRLEENGHEEKNVLSAVDLMVLREKKLAENEGRHPDYFSRTMIDEVFGVIVAGHDTTSTTMSWGVK
LLADNPRIQTKLREALRAGFADAAAEGRSPTIKEVTSAKIHYLDATMEEVLRCGGAALLVDRQATCDTELLGHHIPK
GTVVMCLTRGPSMLKPAFEVDDSKRSKSSQAAKARSWVDEDIGQFKPERWLVGDSGAAPCEFDQQAGPSIAFGL
GLRGCFGRKLAYLELRILVTLIVWNFELLPCPEELSGYGAKEGLTYKPKDCYVRLRALGKN
>*Exserohilum turcica* Et28A
MFLFGQSPDTPGEIPILGITLGRLVTLVAASLITIWALRSWALPKPIPGIPYRRKATRSILGDIPDMLGATANSDKTYM
EWVQEEMQGMNSPIIQMFIRPLSRPVLVLADFRESQDILMRRSKEWDRSDMLAELLKGLLPGHHLVQRTNHVW
KSHRRLLQDLMSPSFLHNVAAPAIYNSASYLISLWDAKAQLVGDQPFSAQDDIYMAALDAVHAFAFGEKFEHNAT
RSKLESLKGLGPEAIASLQKTNATNGVVKAAEFPEIDLDPVITATLDLTTAVERLQGSPLMRLTWKLMELTPSMRRS
KSIKDKHILRELQRAVDHLTLSDQQQQSDSGDTHQPIRSAVDHMVQREKQLAEKENRAPQYFSHTMMIETFGFVI
GGHETTSTTLLWGLKSLADHQDAQVKLRRSLQALFTAARSENRAPSIEEITSTEIPYLDAVIEEILRHGATSPALDRQA
VVDTQVLGHHVPKGTILLMLTQAQSMRSPGFEIDENCRSQTCQTAKQQGKHRTEWDPLDMNLFKPERWLVPAP
TQPSPEKEDHVDAKYVFDSAAGPMLGFGLGLRGCFGRRLAYLELRILVTLIIWNFELLPCPKELSKYSVKMGVTSRPK
LCYVSLRKVEFA
>*Pestalotiopsis fici* W106-1
MTSSIELASMPVITLEQVRPLGFLFVTLLLCCYLLYQWLLPKPIPGIPYNVESSRKIMGDIPALLREINGTDKTFMDYIIK
QVKSHDSPVIQLFLSPFRKPMVIINDFQESQDLLMRRKEFDRSEFLTDLFGGVSPEHHIMKPTNSSWKAHRRLLQDL
MSPPFLHQVAGPNLYSNVNNLISLWNFKADVAQERPFSATDDIYGAALDAIFAFTFGKSFKSNATAPNMHLLQGLS
AQDISKLRGDGKDHDKPLEFPQAPRDKAVTATFDVAHAIEQVNGKPFAKLIWKYVVARKDRFSTALRLKNEYINDE
LKAAVARIQNADSDVRSAVDLMVQRETKIAAKDGRAPDYFSQVMMDETFGFVVAGHETTSTTILWGMKILAANP
RSQTKLRETLRASYSEAFKTKRSPTIDEITGTDIPYLDAVMEEILRCGGTVPGVDRQATCDTEILGYPIKKGTVVLCSGR
GASMLEPGFDIDDSKRSETSRLAKTENRIRAWGNDDIGAFKPERWLVAAPSSEDSTETAYHFDSTAGPQLAFGLGL
RSCFGRRLAYLELRILLTMIVWNFELLPCPEDLAGFSAIAGITYKPRQCYVRLRKAI
>*Phialocephala subalpina* UAMH 11012
MANFSDFIAYLRQYFPLSPLNSLTIITGTISFYLLYRRALPKPIPGIPYNEFSARKFLGDVPAMVSHISQTDGTFVTYLM
STMKSLNAPLIQVFIQPMSKPLLVLADFRETEDLMLRRKDFDRSSDMGDLVKGLVPNHHIQLKTNARWKSQRRLIQ
DLMTPSFLHAVAGPVIHQNASILIELWRTKSRIANGRPWEAAKDINQVALDAVMSFAFGERFRNSATKAALEVVK
GLDEKRVERSGGLDELAEFPPGQIHEVLQAILDLSGTVGEMQGSPLPKLTWAYISRRPRIQKATKIKNEYILNELKHA
VQRREAGEGTIERSAVHHMILREKSFAEKEKRNPDYLSGIMIDEIFGFIFAGHETTSTTICWGLKLLADHPEIQTRVRT
ALQSSYALAKTEGRNPTIQEITGTPIPYLDATMEEMQRFGGTSPIVDREALVDTELLGHHIPKGTNVICLTMGPSMIT
PAFEIDEGRRTASCRAAKEGKEKAWDPEDMGVFIPERWLKDGEFDGAAGPLLAFGLGTRGCYGRRLAYVEMRIIFT
LVLWNFELLQCPPAFSGYKSVLVSTNNPKDCYVRLREVKFNEK
>*Thozetella sp* PMI_491
MDGSFGHFQEYLRLHPFSFAGKILAAALGLALAYLVCLSVYNVFFHPLHSFPGPKLWAMSRIPYTRNSISGRPQKRIL
ELHQTYGPFVRVAPEIVSISHPDAMRDLKGHRKGGKAEHGKDPINVYQFRSSILGANREDHARFRRVMSHGFSAQ
AMIEQQPTIKSYVDLLFKRLHENCADGTQPLDMVRWYNWTTFDIIGDLAFGEPFDCLENASYHPWVSLIFESIKNL
AFWTNASRYRSIAPLLRHFLIPRSLATKLQEHNQLSEMKVRKRLATETDRPDFIGKMVQGPKNKGGDLTFAELSAN
ASILIIAGSETTATLLSGATYLLAKNLRTLATLAEEVRSAFASEDEIDLVNTQHLKYLQAVLEESLRFYPPAPSGQPRKIA
AGGDEIAGRFVPEDAIVETWHWPLYHNPSYFTHPDEFIPERWLGDERFKNDQRDAVQPFSVGPRNCIGRNLAYAE
MRMMLARMVWNFDFKLAEQSKDWYEQNNLFTLWEKGPLYIYLTPRHKT
>*Truncatela angustata* MPI-SDFR-AT-0073
MSSSNAAQYAASVVSLEQLRPYGFFLATLLLCSYLLYQWALPKPIPGIPYNVDSSRKILGDIPALLGEISGTDKTFMNY
VRKQVDQHNSPIIQLFLRPFGKPMVILSDFRESQDILMRRKEFDRSNFMEELFQGVAPEHHIMMPTNDVWKAHR
RLLQDLMSPPFLNHVAGPVLHANVLELIKLWRMKADIAGNRPFSATDDIYDAALDGVFGFAFGEDFEYSATSPNVK

LLEGLSPKDIARLRQSGGGGDDEPLEFMTAPRDDAVSSTFDLAHALEEINGKPLGKLRWNWIVGRKPRTKRALKRR
DDYIYGELKKAVQRMEDRDTSVRSAVDHMMQRETKLAEKDGRAPEYLSPAMADETFGFVIAGHETTSTTVLWAL
KILADNPQPQTKLRRLLQAAHSGAWNEERNPTIDEITGAGIPYLDAVMEEILRCGGTVPAVDREALCDTEVLGYHIP
KGTQVIMSGMGPSMLAPGFDVEEGKRSETCQTAKADGRFKAWRAEDIGKFNPERWLVPLSGVEEEEQQFNSEA
GPQLAFGLGTRGCYGKKLAYLELRILVTMIVWNFELLRCPEDLSSYAAIAGITSKPRKCFVRLRKVL
